# Smad4-dependent suppressor pituitary homeobox 2 promotes PPP2R2A-mediated inhibition of Akt pathway in pancreatic cancer

**DOI:** 10.18632/oncotarget.7158

**Published:** 2016-02-03

**Authors:** Qi Wang, Juanjuan Li, Wei Wu, Ruizhe Shen, He Jiang, Yuting Qian, Yanping Tang, Tingting Bai, Sheng Wu, Lumin Wei, Yi Zang, Ji Zhang, Lifu Wang

**Affiliations:** ^1^ Department of Gastroenterology, Ruijin Hospital Affiliated to Shanghai Jiao Tong University School of Medicine, Shanghai 200025, China; ^2^ State Key Laboratory of Medical Genomics and Sino-French Research Center for Life Sciences and Genomics, Ruijin Hospital Affiliated to Shanghai Jiao Tong University School of Medicine, Shanghai 200025, China

**Keywords:** Pitx2, Smad4, PPP2R2A, pancreatic cancer, carcinogenesis

## Abstract

The importance of Pituitary homeobox 2 (*Pitx2*) in malignancy remains enigmatic, and *Pitx2* has not been previously implicated in pancreatic ductal adenocarcinoma (PDAC). In this study, we performed gene expression profiling of human PDAC tissues and identified *Pitx2* as a promising candidate. *Pitx2* expression was decreased from 2.6- to 19-fold in human PDAC tissues from microarray units. Immunochemistry staining showed that *Pitx2* expression was moderate to intense in normal pancreatic and pancreatic intraepithelial neoplastic lesions, whereas low in human PDAC tissues. The *Pitx2* levels correlated with overall patient survival post-operatively in PDAC. Induction of *Pitx2* expression partly inhibited the malignant phenotype of PDAC cells. Interestingly, low *Pitx2* expression was correlated with *Smad4* mutant inactivation, but not with *Pitx2* DNA-methylation. Furthermore, Smad4 protein bound to *Pitx2* promoter and stimulated *Pitx2* expression in PDAC. In addition, Pitx2 protein bound to the promoter of the protein phosphatase 2A regulatory subunit B55α (*PPP2R2A*) and upregulated *PPP2R2A* expression, which may activate dephosphorylation of Akt in PDAC. These findings provide new mechanistic insights into *Pitx2* as a tumor suppressor in the downstream of *Smad4*. And Pitx2 protein promotes *PPP2R2A* expression which may inhibit Akt pathway. Therefore, we propose that the Smad4-Pitx2-PPP2R2A axis, a new signaling pathway, suppresses the pancreatic carcinogenesis.

## INTRODUCTION

Microarray has been used to further understand the molecular pathogenesis of carcinogenesis and explore differentially expressed genes which may be potential therapeutic targets [1, 2]. In this paper, microarray analysis was performed to identify potential markers correlated with the known suppressors *Smad4*, *p16*, *Tp53* and *BRCA2,* which are most frequently inactivated in pancreatic ductal adenocarcinoma (PDAC). Using gene microarray, we identified pituitary homeobox 2 (*Pitx2*) as a promising candidate marker.

Previous studies showed that *Pitx2* was a paired-like homeodomain transcription factor that was identified as one of the genes responsible for Axenfeld Rieger syndrome [3], and was induced by the Wnt-β-catenin pathway and TGF-β1-FGF9 pathway in cell proliferation during development [4, 5].

Recently, low *Pitx2* expression was reported in patients with breast cancer [6, 7], prostate cancer [8, 9] and colon cancer [10], and it was considered as a potential predictor of poor clinical outcomes, which were correlated with its hypermethylation. Hirose *et al.* also reported that *Pitx2* levels were inversely correlated with *in vitro* colon cancer cell growth and invasion [10]. According to these observations, *Pitx2* may suppress carcinogenesis. However, interestingly, several studies showed that *Pitx2* may promote tumor progression, and it was considered to be a potential oncogene in thyroid cancer [11, 12], prostate cancer [13] and ovarian cancer [14, 15]. These studies indicate the diverse and complex functions of *Pitx2* in carcinogenesis.

The regulatory mechanism of *Pitx2* as an oncogene had also been partly discovered in ovarian cancer, which was associated with the Wnt pathway [14, 15]. However, the regulatory mechanism of *Pitx2* as a tumor suppressor is still unclear, and *Pitx2* has not been previously implicated in PDAC.

In this study, using microarray analysis, we found that *Pitx2* expression was significantly downregulated in PDAC tissues and decreased *Pitx2* expression was correlated with *Smad4* inactivation, but not with *Pitx2* DNA-methylation. Furthermore, *Pitx2* may act as a downstream suppressor in the TGF-β1-Smad4 pathway, and promote the protein phosphatase 2A regulatory subunit B55α (*PPP2R2A*) expression which may inhibit the Akt pathway in pancreatic carcinogenesis.

## RESULTS

### Gene expression profiling for identification of the potential markers correlated with known suppressors *Smad4*, *p16*, *Tp53* and *BRCA2* in human PDAC

To identify genes with potential roles in the progression of PDAC, we performed gene expression profiling of the microdissected tissues of human PDAC using 4 × 44K Agilent microarrays. To investigate mRNAs with statistically significant differences in expression, heatmap and volcano plot filtering on eight human PDAC tissues and paraneoplastic tissues were performed (Supplementary Figure S1A and S1B), and thirty-five upregulated genes and twenty-nine downregulated genes (≥2-fold, *p*-value< 0.05) were identified (Supplementary Table S1).

**Figure 1:**
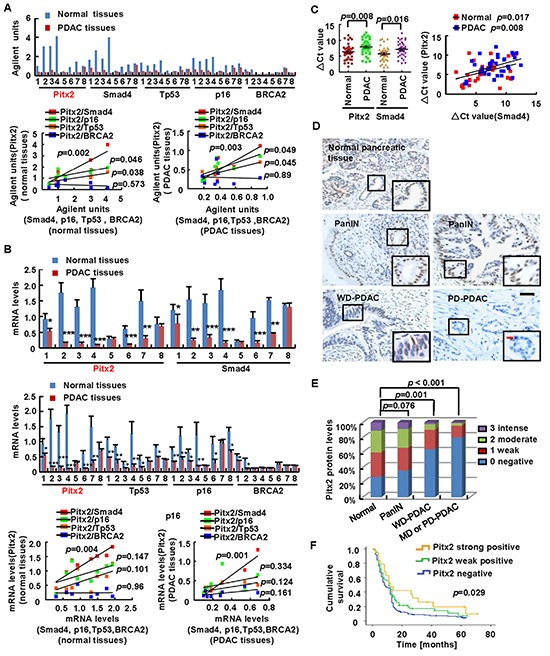
Gene expression profiling for identification of biomarkers and clinical significance of *Pitx2* **A, B.** Pearson's correlation analysis explored the correlation of gene expression between *Pitx2* and suppressors *Smad4*, *Tp53*, *p16* or *BRCA2* according to microarray units (A) and real-time PCR (B) in eight human PDAC and paraneoplastic tissues. **C.** Pearson's correlation test analyzed the correlation between *Pitx2* and *Smad4* according to real-time PCR in thirty-six human PDAC and paraneoplastic tissues. **D, E.** Immunohistochemistry staining of Pitx2 was summarized in the histogram. black bar scale = 50 μm, red bar scale = 10 μm. WD-PDAC, MD-PDAC, PD-PDAC: well, moderately, poorly-differentiated adenocarcinoma. **F.** Kaplan-Meier survival curves of Pitx2-strong positive (grade 2 or 3), Pitx2-weak positive (grade 1) and Pitx2-negative (grade 0) patients were performed using the log-rank test.

We aimed to identify potential markers correlated with the known suppressors *Smad4*, *p16*, *Tp53* or *BRCA2* with the most frequently inactivated mutation in PDAC, among the sixty-four differentially expressed genes. Interestingly, according to the microarray results, *Pitx2* expression was strongly correlated with *Smad4*, *p16* and *Tp53* expression using Pearson's correlation test (Supplementary Figure S1C), and it significantly decreased from 2.65- to 19.62-fold in six of the eight PDAC tissues compared to the control (Figure 1A). We observed that in the PDAC and paraneoplastic tissues, the *Pitx2* levels were significantly correlated with the *Smad4* levels (PDAC: *p* = 0.003; paraneoplastic tissues: *p* = 0.002), *p16* levels (PDAC: *p* = 0.049; paraneoplastic tissues: *p* = 0.046), and *Tp53* levels (PDAC: *p* = 0.045; paraneoplastic tissues: *p* = 0.038). However, the *Pitx2* levels did not show a correlation with the *BRCA2* levels in PDAC (*p* = 0.896) or paraneoplastic tissues (*p* = 0.573) (Figure 1A).

Real-time PCR analysis of *Pitx2*, *Smad4*, *p16*, *Tp53* and *BRCA2* expression showed that in six of the eight PDAC tissues, *Pitx2, Smad4, p16*, and *Tp53* levels were significantly decreased from 1.71- to 19.20-fold, 1.55- to 10.83-fold, 2.01- to 6.54-fold, and 1.75- to 6.75-fold, respectively. However, *BRCA2* levels were decreased from 1.48- to 4.32-fold in two tissues, compared to the control. The PCR results were consistent with the microarray data (Figure 1B).

To further examine whether *Pitx2* expression had a significant correlation with *Smad4*, *p16* or *Tp53* expression, Pearson's correlation test was performed on the PCR data. As shown in Figure 1B, *Pitx2* expression was significantly correlated with *Smad4* expression in PDAC (*p* = 0.004) and paraneoplastic tissues (*p* = 0.001). However, *Pitx2* expression was not correlated with *p16* expression in PDAC (*p* = 0.394) or paraneoplastic tissues (*p* = 0.144) and with *Tp53* expression in PDAC tissues (*p* = 0.119) or paraneoplastic tissues (*p* = 0.135). We then evaluated the *Pitx2* and *Smad4* levels in an increased sample size of thirty-six PDAC and paraneoplastic tissues by real-time PCR. Pearson's correlation analysis revealed that *Pitx2* expression had a strong correlation with *Smad4* expression in the thirty-six PDAC (*p* = 0.008) and paraneoplastic tissues (*p* = 0.017) (Figure 1C).

These data demonstrate that the significant decrease in the *Pitx2* levels may be correlated with decrease in the *Smad4* levels in PDAC.

### Dynamic alteration and clinical significance of *Pitx2* expression in human PDAC tissues

To determine whether the differential expression of *Pitx2* was clinically significant, immunohistochemistry staining was performed to assess the Pitx2 levels in 256 PDAC tissues (including 162 pancreatic intraepithelial neoplastic lesions[PanIN]) and 256 paraneoplastic tissues. The percentage of stained cells was scored from grade 0 to 3. Weak, moderate, and intense Pitx2 nuclear staining was observed in 83, 75 and 28 of the 256 paraneoplastic pancreatic tissue samples, respectively. Weak, moderate, and intense Pitx2 nuclear staining was detected in 104 of the 162 PanIN lesions. In contrast, no Pitx2 nuclear staining was observed in 59 of the 92 well-differentiated adenocarcinoma (WD-PDAC) tissues and in 131 of the 164 moderately- or poorly-differentiated adenocarcinoma (MD-PDAC or PD-PDAC) tissue samples (Figure 1D and 1E). The Pitx2 levels did not show significant downregulation in PanIN lesions compared to paraneoplastic pancreatic tissues (*p* = 0.076), however, were sharply decreased in WD-PDAC tissues (*p* = 0.001) and MD or PD-PDAC tissues (*p* < 0.001) compared to paraneoplastic pancreatic tissues (Figure 1E).

Furthermore, the PDAC samples were divided into three groups for clinicopathological evaluation according to the Pitx2 levels: strong positive Pitx2 staining (grade 2 or 3), weak positive Pitx2 staining (grade 1) and negative Pitx2 staining (grade 0). Kaplan-Meier analysis showed that the postoperative median survival was 24.97 months for patients with strong positive Pitx2 staining, 17.72 months for patients with weak positive Pitx2 staining and 13.1 months for patients with negative Pitx2 staining (*p* = 0.029) (Figure 1F). These data demonstrate that *Pitx2* expression was sharply decreased in PDAC tissues, which is probably correlated with the poor prognosis in PDAC patients.

### *Pitx2* overexpression may attenuate the malignancy of PDAC cells *in vitro* and *in vivo*

**Figure 2:**
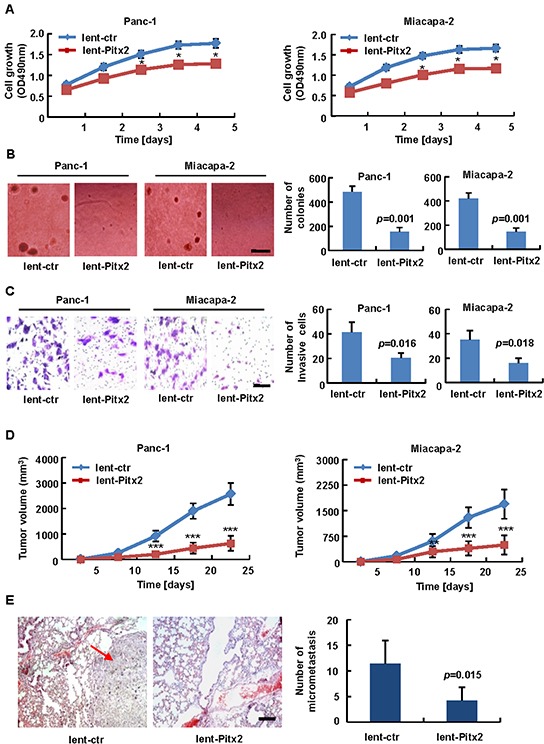
Biological role of *Pitx2* in pancreatic carcinogenesis After lentivirus-mediated Pitx2-overexpression vector or the control (lent-Pitx2 or lent-ctr) was transduced into PDAC cells, MTT assay **A.** soft agar assay **B.** and Matrigel invasion assay **C.** showed the anchorage-dependent, anchorage-independent growth ability and tumor invasion rate (bar scale = 500 μm). The subcutaneous tumorigenic ability **D.** and lung metastasis after vein injection **E.** of tumor cells were measured (*n* = 5). HE-stained sections of lung: bar scale = 200 μm; red arrow, metastatic nodule. *, *p* < 0.05; **, *p* < 0.01; ***, *p* < 0.001.

As the function of *Pitx2* in PDAC is unclear, we tried to determine its biological role. Firstly, lentivirus-mediated *Pitx2*-overexpression vector or the control was stably transduced into Panc-1 cells (Panc-1-lent-Pitx2 or Panc-1-lent-ctr) and Miacapa-2 cells (Miacapa-2-lent-Pitx2 or Miacapa-2-lent-ctr). MTT assay showed the growth rate of Panc-1 or Miacapa-2 cells with *Pitx2* overexpression was lower than the control (Figure 2A). Soft agar assay showed that the Panc-1-lent-ctr and Miacapa-2-lent-ctr cells formed 484.7 ± 45.5 and 431 ± 46 colonies, respectively, while the Panc-1-lent-Pitx2 and Miacapa-2-lent-Pitx2 cells only formed 155.7 ± 33.5 (*p* = 0.001) and 146.7 ± 29.7 (*p* = 0.001) colonies post twenty-six days, respectively (Figure 2B). Invasion assay showed that the overexpression of *Pitx2* promoted the invasive ability of Panc-1 (41.3 ± 8 vs. 20.3 ± 4 of stained cells, *p* = 0.016) and Miacapa-2 cells (35 ± 7.5 vs. 16 ± 4 of stained cells, *p* = 0.018) (Figure 2C). To determine whether *Pitx2* inhibited tumorigenicity and micrometastasis of PDAC cells *in vivo*, Panc-1-lent-Pitx2, Miacapa-2-lent-Pitx2 cells or the control were injected into the subcutis or the tail vein of five nude mice each. The results demonstrated that overexpression of *Pitx2* resulted in a significant decrease in tumorigenicity (Figure 2D and Supplementary Figure S2A). Moreover, quantification of micrometastasis in lung tissues and H&E staining showed that *Pitx2* overexpression inhibited the *in vivo* micrometastasis of PDAC cells (11.4 ± 4.5 vs. 4.2 ± 2.6, *p* = 0.015) (Figure 2E and Supplementary Figure S2B).

These data suggest that *Pitx2* acted as a potential tumor suppressor in PDAC cells.

### *Pitx2* is a target gene of *Smad4* in PDAC cells

Next, we aimed to explore the underlying mechanism of low *Pitx2* expression in PDAC. To determine whether *Pitx2* DNA-hypermethylation caused low *Pitx2* expression in PDAC, the *Pitx2* DNA-methylation levels and *Pitx2* mRNA levels were detected in human PDAC cell lines HPAF-II, Miacapa-2, Panc-1, BxPC-3, Capan-1, AsPC-1 and CFPAC-1. Human breast cancer cell line MCF-7 with *Pitx2* DNA-hypermethylation was used as the positive control [6]. The results showed that relative *Pitx2* DNA-methylation levels were extremely low, between 0.14% and 6.67%, in PDAC cells compared to MCF-7 cells (58.1%), and *Pitx2* mRNA levels in PDAC cell lines were low, from 0.03% to 7.16 % (Supplementary Figure S3A). We also compared the *Pitx2* DNA-methylation levels in thirty-six fresh human PDAC and paraneoplastic tissues. There was no significant difference of *Pitx2* DNA-methylation between the two groups (Supplementary Figure S3B). Importantly, we found that the *Pitx2* DNA-methylation levels did not correlate with the *Pitx2* mRNA levels in thirty-six human PDAC tissues using Pearson's correlation test (*p* = 0.275) (Supplementary Figure S3C). These results reveal that factors other than *Pitx2* DNA-hypermethylation lead to low *Pitx2* expression in PDAC.

**Figure 3:**
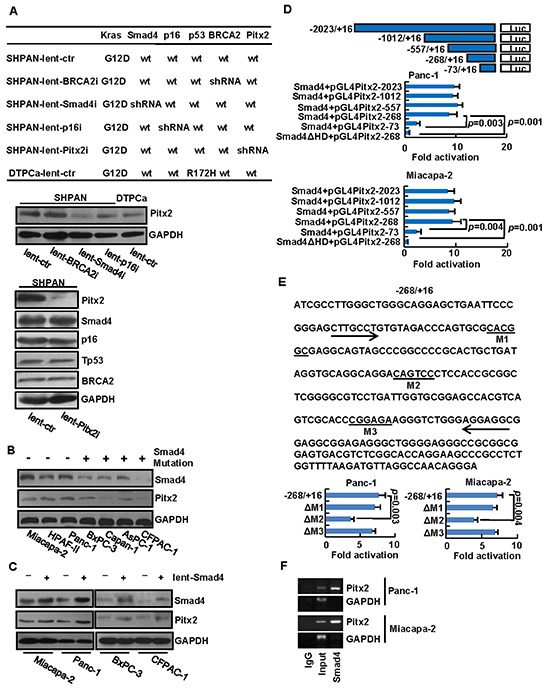
Identification of *Pitx2* gene as a direct target gene of *Smad4* **A.** After lentivirus-mediated shRNA vector or the control was transfected into SHPAN and DTPCa cells, namely SHPAN-lent-i or SHPAN (DTPCa)-lent-ctr, the correlation between *Pitx2* and *Smad4*, *Tp53*, *p16* or *BRCA2* was confirmed. **B.** Pitx2 and Smad4 were detected in PDAC cell lines with or without *Smad4* mutant inactivation. **C.** After *Smad4* overexpression (lent-Smad4) in PDAC cells, the Pitx2 protein levels were detected. **D.** The map illustrated the *Pitx2* promoter region. *Pitx2* promoter activity after cotransfection with Smad4 plasmid and each of the promoter constructs were detected. **E.** Analysis of the nucleotide sequence of the −268/+16 construct with three potential Smad4 binding sites M1, M2, and M3. M1, M2, and M3 mutations were introduced. **F.** ChIP analysis showed that Smad4 bound to the *Pitx2* promoter.

To confirm the correlation of *Pitx2* with *Smad4*, *Tp53*, *p16* and *BRCA2* in human PDAC and paraneoplastic tissues, lentivirus-mediated *Smad4*, *p16*, *BRCA2*, *Pitx2*-shRNA vector or the control were stably transduced into SHPAN cells or DTPCa cells [16, 17]. The knockdown effects of Smad4, p16 and BRCA2 were shown in Supplementary Figure S4 and the pairs of shRNA with the best knockdown effect were used, respectively. The genotype of the cell lines was shown in Figure 3A. We found that the Pitx2 levels were significantly decreased in SHPAN-lent-Smad4i cells compared to the control, whereas knockdown of *Pitx2* did not alter the protein levels of Smad4, p16, Tp53, or BRCA2 in SHPAN cells (Figure 3A). The results indicate that *Pitx2* expression might be correlated with *Smad4* expression in mouse cells.

Next, we investigated whether *Pitx2* expression was correlated with the status of *Smad4* expression in human PDAC cell lines. The Pitx2 levels in BxPC-3, Capan-1, AsPC-1 and CFPAC-1 cells with *Smad4* mutant inactivation were significantly lower than those in Miacapa-2, HPAF-II and Panc-1 cells with wild-type *Smad4* (Figure 3B). To determine whether *Pitx2* expression was correlated with *Smad4*, lentivirus-mediated *Smad4*-overexpression vector or the control was stably transduced into Miacapa-2, Panc-1, BxPC-3 and CFPAC-1 cells, and the Pitx2 levels were significantly upregulated in PDAC cells with *Smad4* overexpression (Figure 3C).

To identify whether Smad4 protein binds to *Pitx2* promoter, we constructed a series of reporter plasmids that cover various lengths of the *Pitx2* promoter from −2023 to +16. Luciferase promoter–reporter assays showed that the effect of the *Smad4* expression vector in Panc-1 and Miacapa-2 cells was maintained in the −1012/+16, −557/+16, and −268/+16 truncated constructs, but lost in the −73/+16 construct (Figure 3D). These results indicate that the regulatory region primarily involved in *Smad4*-mediated *Pitx2* expression was located in the −268/+16 upstream region of the promoter, which contained three putative Smad4-binding sites, namely M1, M2, and M3 (Figure 3E). To validate whether M1, M2 or M3 was Smad4-binding site, the three ones were deleted to abolish Smad4 binding, respectively. The results demonstrated that the ΔM1 and ΔM3 constructs did not significantly inhibit Smad4 binding, but the ΔM2 mutated construct significantly inhibited Smad4 binding compared to the control (Figure 3E). The protein levels of Pitx2 were shown in Supplementary Figure S5A. These results suggest that the putative M2 site in the *Pitx2* promoter may be essential for Smad4-mediated activation. To confirm whether *Smad4* directly binds to *Pitx2*, ChIP analysis was conducted. Primers were designed to amplify the −268/+16 site in the *Pitx2* promoter. ChIP analysis showed that *Pitx2* was markedly detected from the Smad4-immunoprecipitated Panc-1 and Miacapa-2 chromatins, but absent from chromatin immunoprecipitated by the control rabbit immunoglobulin G (Figure 3F).

Taken together, these data suggest that Smad4 protein directly binds to the promoter of *Pitx2* and that ectopic expression of *Smad4* results in an increase in the *Pitx2* levels in PDAC.

### *Pitx2* is a downstream tumor suppressor in the TGF-β-Smad4 pathway in PDAC

**Figure 4:**
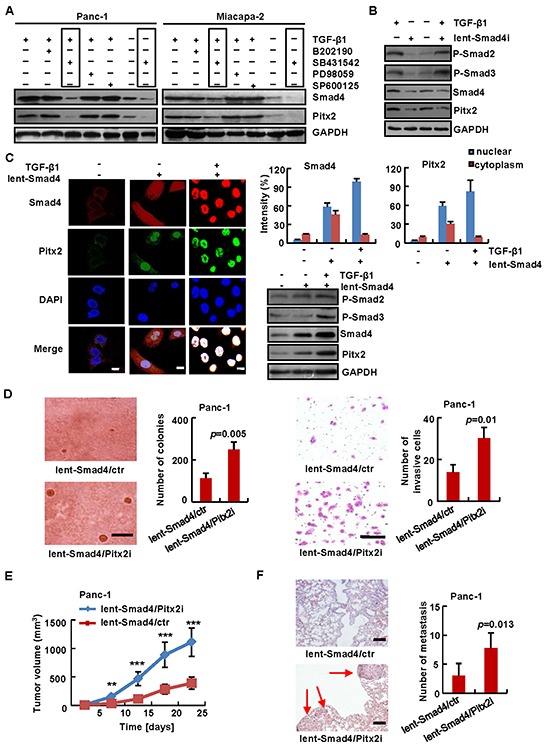
*Pitx2* was a tumor suppressor in the downstream of TGF-β-Smad4 pathway *TGF-β1*-induced *Pitx2* expression was analyzed in the absence or presence of inhibitors **A.** After lentivirus-mediated *Smad4*-shRNA (lent-Smad4i) **B.** or *Smad4-*overexpression (lent-Smad4) **C.** or the control (lent-ctr) vector was transduced into Panc-1 cells, protein levels were analyzed in the absence or presence of TGF-β1 treatment. Lentivirus-mediated *Smad4-*overexpression/*Pitx2*-shRNA (lent-Smad4/Pitx2i) or *Smad4-*overexpression/control-shRNA (lent-Smad4/ctr) vector was stably cotransduced into Panc-1 cells, respectively, soft agar (bar scale = 500 μm) and Matrigel invasion assay (bar scale = 50 μm) showed the anchorage-independent growth ability and tumor invasion rate **D.** The subcutaneous tumorigenic ability **E.** and lung metastasis after vein injection **F.** of tumor cells were measured (*n* = 5). HE-stained sections of lung: bar scale = 200 μm; red arrow, metastatic nodule. *, *p* < 0.05; **, *p* < 0.01; ***, *p* < 0.001.

To clarify whether *Pitx2* is a tumor suppressor in the downstream of TGF-β-Smad4 pathway in PDAC, we firstly detected the Pitx2 levels with or without inhibitors against ERK1*/2* (PD98059), p38 (SB202190), TGFβ1-Smads (SB431542), and JNK (SP600125). As shown in Figure 4A, only SB431542 resulted in a decrease of *Pitx2* expression in Panc-1 and Miacapa-2 cells with or without TGF-β1 treatment, whereas treatment with PD98059, SB202190, or SP600125 had no effect. Then, we examined whether TGF-β1 would promote *Pitx2* expression. Lentivirus-mediated *Smad4*-shRNA vector or the control was stably transduced into Panc-1 cells, and the Pitx2 levels in the cells with or without TGF-β1 treatment were determined. The results showed that TGF-β1 may result in an increase in the Pitx2 levels in the presence of Smad4; however, TGF-β1 may not alter the Pitx2 levels in the absence of Smad4 (Figure 4B). In addition, lentivirus-mediated *Smad4*-overexpression vector or the control was stably transduced into Panc-1 cells, and immunofluorescence staining and immunoblotting analysis were performed to measure the levels and locations of Pitx2 and Smad4 with or without TGF-β1 treatment in the cells. Nuclear and cytoplasm Pitx2 and Smad4 staining was sharply increased with overexpression of *Smad4*, and treated with TGF-β1 further increased the Pitx2 and Smad4 levels and induced Pitx2 and Smad4 nuclear import (Figure 4C). These data show that *Pitx2* expression may be altered by TGF-β1 in the presence of Smad4.

To further determine whether *Pitx2* was a potential suppressor in the downstream of *Smad4*, we examined whether knockdown of *Pitx2* altered the phenotypes of PDAC cells with *Smad4* restoration. Lentivirus-mediated *Smad4-*overexpression/*Pitx2*-shRNA (Panc-1-lent-Smad4/Pitx2i) or *Smad4*-overexpression/control-shRNA (Panc-1-lent-Smad4/ctr) vector was stably cotransduced into Panc-1 cells. Soft agar assay showed that Panc-1-lent-Smad4/Pitx2i cells formed 248.7 ± 35.6 colonies, while Panc-1-lent-Smad4/ctr cells only formed 113.7 ± 23.5 colonies after twenty-six days (*p* = 0.005) (Figure 4D). The invasion assay showed that knockdown of *Pitx2* promoted the invasive ability of Panc-1 cells with Smad4-overexpression (14 ± 3.6 vs. 30.3 ± 5 of stained cells, *p* = 0.01) (Figure 4D). Moreover, Panc-1-lent-Smad4/Pitx2i or Panc-1-lent-Smad4/ctr cells were injected into the subcutis or tail vein of nude mice. The results showed that the tumorigenicity of Panc-1-lent-Smad4/Pitx2i cells was increased compared to Panc-1-lent-Smad4/ctr cells (Figure 4E and Supplementary Figure S6A). Moreover, quantification of micrometastasis in lung tissues and H&E staining demonstrated that knockdown of *Pitx2* enhanced micrometastasis of Panc-1 cells with Smad4-overexpression (7.8 ± 2.6 vs. 3 ± 2.1, *p* = 0.013) (Figure 4F and Supplementary Figure S6B). These results indicate that knockdown of *Pitx2* promoted the tumorigenicity and micrometastasis of Panc-1 cells with Smad4-overexpression.

These data suggest that *Pitx2* acted as a potential suppressor downstream of the TGF-β1-Smad4 pathway in PDAC cells.

### *Pitx2* may promote Akt dephosphorylation via upregulation of *PPP2R2A* in PDAC

**Figure 5:**
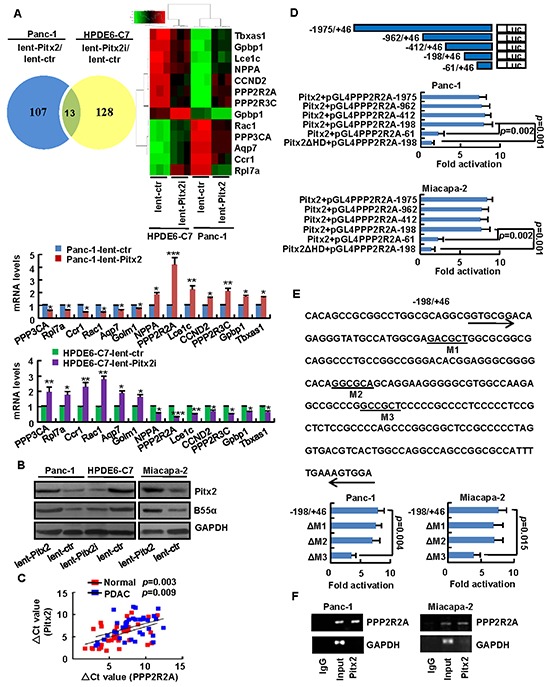
*Pitx2* may stimulate *PPP2R2A* expression in PDAC cells Lentivirus-mediated *Pitx2-*overexpression vector or the control was stably transduced into Panc-1 cells (lent-Pitx2 or lent-ctr), and *Pitx2-*shRNA or the control was stably transduced into human pancreatic duct epithelial cell line HPDE6-C7 (lent-Pitx2i or lent-ctr). **A.** Venn diagram and the heatmap showed overlap of differentially expressed genes. *PPP2R2A* was the most significantly differentially expressed gene among the overlapped thirteen genes (*, *P* < 0.05; **, *p* < 0.01; ***, *p* < 0.001). **B.** Ectopic *Pitx2* increased B55α levels and knockdown of *Pitx2* decreased B55α levels. **C.** *Pitx2* expression in thirty-six human PDAC tissues samples were correlated with *PPP2R2A* expression according to PCR data by Pearson's correlation test. **D.** The map illustrated the *PPP2R2A* promoter region. *PPP2R2A* promoter activity after cotransfection with Pitx2 plasmid and each of the promoter constructs were detected. **E.** Analysis of the nucleotide sequence of the −198/+46 construct with three potential Pitx2 binding sites M1, M2, and M3. M1, M2, and M3 mutations were introduced. **F.** ChIP analysis showed that Pitx2 bound to the *PPP2R2A* promoter.

We next identified the potential downstream markers of *Pitx2*. Lentivirus-mediated *Pitx2*-overexpresion vector or the control was stably transduced into in Panc-1 cells and lentivirus-mediated *Pitx2-*shRNA vector or control was stably transduced into in HPDE6-C7 cells. Microarray was performed on cell lines in both groups, and genes were considered differentially expressed if expression values changed by at least 2-fold. Microarray analysis identified 107 differentially expressed genes in Panc-1 cells with *Pitx2*-overexpression and 128 differentially expressed genes in HPDE6-C7 cells with *Pitx2*-shRNA, compared to the control (Figure 5A, Supplementary Table S2 and Supplementary Table S3). The thirteen differentially expressed genes that overlapped between the two cell groups were chosen for validation by real-time PCR. According to the PCR data, the protein phosphatase 2A regulatory subunit B55α (*PPP2R2A*) was the most significantly differentially expressed gene among the thirteen genes in both groups of cells, which were consistent with the microarray data. Induction of *Pitx2* upregulated B55α (PPP2R2A) levels in Panc-1 and Miacapa-2 cells, while knockdown of *Pitx2* downregulated B55α levels in HPDE6-C7 cells (Figure 5B). To testify whether *Pitx2* was correlated with *PPP2R2A*, *Pitx2* and *PPP2R2A* levels were detected in thirty-six fresh PDAC or paraneoplastic tissue samples. Pearson's correlation analysis showed that *Pitx2* expression was significantly correlated with *PPP2R2A* expression in thirty-six PDAC or paraneoplastic tissues (Figure 5C). To identify whether Pitx2 protein binds to *PPP2R2A* promoter, a series of reporter plasmids that cover various lengths of the *PPP2R2A* promoter from −1975 to +46 were constructed. Luciferase promoter–reporter assays showed that the effect of the *Pitx2* expression vector in Panc-1 and Miacapa-2 cells was maintained in the −1962/+46, −412/+46, and −198/+46 truncated constructs, but lost in the −61/+46 construct, which demonstrated that the regulatory region primarily involved in Pitx2-mediated *PPP2R2A* expression was located in the −198/+46 upstream region of the promoter (Figure 5D). The −198/+46 region may contain three putative Pitx2-binding sites, namely M1, M2, and M3 (Figure 5E). To testify whether M1, M2 or M3 was Pitx2-binding site in Panc-1 and Miacapa-2 cells, the three ones were deleted to abolish Pitx2 binding, respectively. Expression of the ΔM1 and ΔM2 constructs did not significantly alter Pitx2 binding, but expression of the ΔM3 construct resulted in a significant decrease in Pitx2 binding (Figure 5E). The protein levels of Pitx2 were shown in Supplementary Figure S5B. The results indicate that the putative M3 site in the *PPP2R2A* promoter may be essential for Pitx2-mediated activation. To confirm whether Pitx2 protein directly binds to *PPP2R2A* promoter, ChIP analysis was conducted. Primers were designed to amplify the −198/+46 site in the *PPP2R2A* promoter. ChIP analysis showed that *PPP2R2A* was markedly detected from the Pitx2-immunoprecipitated Panc-1 and Miacapa-2 chromatins, but absent from chromatin immunoprecipitated by the control rabbit immunoglobulin G (Figure 5F). Taken together, these data suggest that Pitx2 protein directly binds to the promoter of *PPP2R2A* and that ectopic expression of *Pitx2* results in an increase of *PPP2R2A* levels in PDAC.

**Figure 6:**
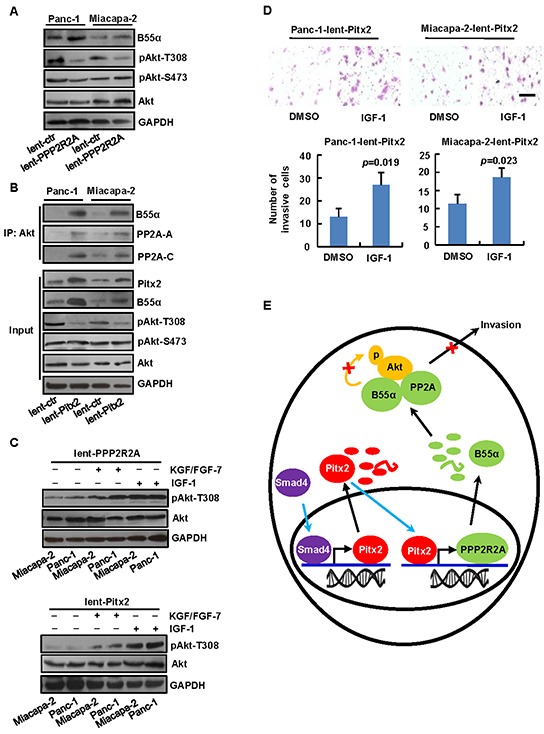
The Akt pathway involved in *Pitx2*-induced B55α expression **A.** Lentivirus-mediated *PPP2R2A-*overexpression vector or the control was stably transduced into Panc-1 (lent-PPP2R2A or lent-ctr) and Miacapa-2 (lent-PPP2R2A or lent-ctr). B55α may dephosphorylate of Akt at Thr-308 in PDAC cells. **B.** After Lentivirus-mediated *Pitx2-*overexpression vector or the control was stably transduced into Panc-1 and Miacapa-2 cells (lent-Pitx2 or lent-ctr), immunoprecipitation using anti-Akt antibody and the detection of protein levels were performed. **C.** Akt activators IGF-1 or KGF/FGF-7 stimulated the phosphorylation at Thr-308 of Akt in Panc-1 and Miacapa-2 cells with PPP2R2A or Pitx2-overexpression **D.** Invasion assay was performed in Panc-1 and Miacapa-2 cells with *Pitx2* overexpression in the presence or absence of IGF-1 (bar scale = 50μm). **E.** Simplified schematic diagram of the Smad4-Pitx2-PPP2R2A pathway in PDAC cells was shown. Smad4 protein binds to *Pitx2* promoter and stimulates *Pitx2* expression. Pitx2 protein binds to *PPP2R2A* promoter and promotes transcription of *PPP2R2A*, which lead to accumulation of B55α in the cytoplasm and results in the dephosphorylation of Akt.

To determine whether *PPP2R2A* expression inhibited Akt in PDAC, lentivirus-mediated *PPP2R2A-*overexpression vector or the control was stably transduced into Panc-1 and Miacapa-2 cells. Immunoblotting analysis indicated that B55α (PPP2R2A) promoted dephosphorylation at Thr-308 of Akt in Panc-1 and Miacapa-2 cells (Figure 6A). As shown in Figure 5B that *Pitx2* overexpression upregulated B55α levels, we then determined whether *Pitx2* promoted B55α expression, which may inhibit Akt pathway in PDAC cells. We immunoprecipitated endogenous Akt by an anti-Akt antibody from the Panc-1 and Miacapa-2 cells and examined the association of the protein phosphatase 2A (PP2A) complexes in the immunoprecipitated Akt. The interaction between Akt and B55α, PP2A-C, or PP2A-Aα subunit was markedly increased in the cells with *Pitx2* overexpression, which suggests the possible effects of Pitx2-induced B55α on Akt phosphorylation. In addition, Pitx2-induced B55α mainly binds to the PP2A holoenzyme to catalyze dephosphorylation at Thr-308 of Akt but not at Ser-473 in Panc-1 and Miacapa-2 cells (Figure 6B).

To confirm whether inhibition of the Akt pathway by Pitx2-induced B55α was correlated with cell invasion, Akt activators IGF-1 and KGF/FGF-7 were used. The results showed that IGF-1 and KGF/FGF-7 may stimulate the phosphorylation at Thr-308 of Akt in Panc-1 and Miacapa-2 cells with *PPP2R2A* or *Pitx2*-overexpression, and that IGF-1 had more significant effect (Figure 6C). Moreover, the invasion of Panc-1 and Miacapa-2 cells with *Pitx2*-overexpression was significantly activated by IGF-1 (Figure 6D).

Taken together, these results demonstrate that B55α (PPP2R2A) induced by Pitx2 inhibited the Akt pathway, which may be responsible for suppressing PDAC cell invasion. A simplified schematic diagram of the genetic pathway was summarized in Figure 6E.

## DISCUSSION

Recently, Pitx2 was found to play diverse and complex roles in carcinogenesis. Because of its hypermethylation, low *Pitx2* expression had been reported in patients with breast cancer [6, 7], prostate cancer [8, 9], and colon cancer [10], which was considered to be a potential predictor of poor clinical outcomes. Moreover, Hirose *et al.* reported that *Pitx2* expression was inversely correlated with colon cancer cell growth and invasion *in vitro* [10], which suggests that *Pitx2* may act as a tumor suppressor in colon cancer. Interestingly, Huang *et al.* investigated that knockdown of *Pitx2* in human thyroid cancer cells significantly reduced cell proliferation [11]. Vela *et al* reported that *Pitx2* specific shRNA inhibited prostate cancer cell migration toward bone cell derived chemoattractant [13]. These studies suggest that *Pitx2* may act as an oncogene in the cancer types.

Using microarray analysis, we tried to identify the potential markers correlated with *Smad4*, *p16*, *Tp53* and *BRCA2* in human PDAC tissues. We found that *Pitx2* expression was sharply decreased in PDAC tissues, and was significantly correlated with *Smad4* expression. As *Pitx2* has not been previously implicated in PDAC, we were interested in exploring the expression and function of *Pitx2* in PDAC. We found that low *Pitx2* expression was correlated with poor prognosis in PDAC patients, and *Pitx2* may act as a tumor suppressor in PDAC.

Low *Pitx2* expression had been reported correlated with its hypermethylation status [6, 7]. To examine whether low *Pitx2* levels in PDAC tissues were correlated with its DNA-hypermethylation, we examined the *Pitx2* DNA-methylation levels in PDAC. Interestingly, we identified that the *Pitx2* DNA-methylation levels in PDAC were extremely low.

Previous reports had indicated the role of *Pitx2* as an oncogene that was associated with the Wnt pathway [14, 15]. However, the regulatory mechanism of *Pitx2* as a tumor suppressor is still unclear. Therefore, we tried to explore the underlying mechanism in PDAC. Remarkably, we found *Pitx2* levels were significantly correlated with *Smad4* levels in PDAC tissues according to microarray units and following verification. Luciferase and ChIP analysis indicated that Smad4 protein bound to Pitx2 promoter and stimulated *Pitx2* expression. Moreover, TGF-β1 may stimulate *Pitx2* expression and induces Pitx2 nuclear import in the presence of Smad4. Taken together, these findings suggest that *Pitx2* may be a downstream suppressor in the TGFβ1-Smad4 pathway.

Furthermore, we tried to elucidate how *Pitx2* regulated downstream targets during pancreatic carcinogenesis. CyclinD2 and CyclinA1 had been reported to be downstream targets of *Pitx2* that promote thyroid carcinogenesis [11, 12]. In our study, we identified that *Pitx2* expression stimulated B55α (*PPP2R2A*) expression. Previous studies demonstrated that B55α was a regulatory subunit of PP2A, and PP2A may inhibit Akt pathway[18, 19]. We previously showed that B55α may cause dephosphorylation both at Thr-308 and Ser-473 of Akt in mouse cells [20]. Consistent with previous reports [21], in this study, we found that B55α bound to the PP2A holoenzyme to catalyze dephosphorylation at Thr-308 of Akt but not at Ser-473 in human PDAC cells, which may be responsible for the inhibition of PDAC cell invasion.

In summary, our findings show that Smad4 protein may stimulate the transcription of *Pitx2* and *Pitx2* may promote B55α (*PPP2R2A*) expression which may inhibit Akt pathway in PDAC. We propose that the Smad4-Pitx2-PPP2R2A axis, a new signaling pathway, suppresses the pancreatic carcinogenesis.

## MATERIALS AND METHODS

### Patients and tissue samples

Two hundred and fifty-six PDAC and paraneoplastic tissues were obtained from Ruijin Hospital (Shanghai, China). None of the patients underwent radiotherapy or chemotherapy before surgery. The tissues were embedded in paraffin wax for analysis. Histological diagnoses were performed by two independent senior pathologists. Thirty-six fresh PDAC tissues and paraneoplastic tissues obtained after surgery were frozen for the following experiments.

### Cell lines

Low-passage-number cells (P8) of the preinvasive pancreatic ductal cell line SHPAN isolated from *Pdx-1-Cre;LSL-Kras^G12D/+^* mutant mice [16] and the PDAC cell line DTPCa isolated from the *Pdx1-Cre; LSL-Kras^G12D/+^*; LSL-Tp53*^R172H/+^* compound mutant mice [17] were employed. Human PDAC cell lines with wild-type *Smad4* (Panc-1, Miacapa-2, HPAF-II) and with *Smad4* mutant inactivation (AsPC-1, BxPC-3, Capan-1, CFPAC-1) were purchased from the American Type Culture Collection [22, 23]. Panc-1 and HPAF-II, were grown in DMEM; AsPC-1 and BxPC-3 cells were grown in RPMI 1640 medium; Miacapa-2 cells were grown in McCoy's 5a Medium; Capan-1 and CFPAC-1 cells were grown in Iscove's Modified Dulbecco's Medium. A human pancreatic duct epithelial cell line HPDE6-C7 was cultured in RPMI 1640 medium [24]. All cells were cultured in the above-mentioned media supplemented with 10% heat-inactivated fetal bovine serum at 37°C with 5% CO_2_.

### Reagents

The MEK inhibitor PD98059 and TGF-β1-Smad4 inhibitor SB431542 were purchased from Calbiochem. A selective inhibitor of p38 mitogen-activated protein kinase SB202190 and JNK inhibitor SP600125 were purchased from Biomol. Human TGF-β1 and Akt activators KGF/FGF-7 and IGF-1 were purchased from R&D Systems Inc.

### Gene expression analysis and quantitative real-time PCR

Total RNA was isolated by Trizol, and RNA quality was checked using denaturing agarose gels. For microarray, the cRNA was purified and then hybridized to the 4×44K oligo microarray manufactured by Agilent Technologies. Microarray data were analyzed using an Agilent G2565BA Microarray Scanner. Comparative analysis was done using GeneSpring GX 10 (Agilent Technologies) [2, 25–26]. The experimental data were compared to baseline data. Genes were considered differentially expressed if expression changed by at least two-fold.

### Lentivirus-mediated shRNA or gene overexpression

The shRNA target sequences containing four different sequences of mouse Smad4 (GenBank accession no. NM_008540.2), human Smad4 (GenBank accession no. NM_005359.5), mouse p16 (GenBank accession no. NM_009877.2), mouse BRCA2 (GenBank accession no. NM_001081001.1), human Pitx2 (GenBank accession no. NM_153426.2) and mouse Pitx2 (GenBank accession no. NM_001042504.1) were selected for shRNA interference. Lentivirus-mediated overexpression of human Pitx2, human Smad4 and human PPP2R2A (GenBank accession no. NM_002717.3) were constructed using the pGLV5-EF1a-GFP vectors (GenePharma, Shanghai, China). Cells were grown in culture medium with 10 μg/ml puromycin for selection of stable cells [25, 27]. Primers were shown in Supplementary Table S4.

### Methylation-specific real-time PCR and quantitative real-time PCR

Genomic DNA from seven human pancreatic cancer cell lines and 36 human PDAC tissues were extracted with the DNeasy Tissue kit (Qiagen). Methylation-specific PCR (MSP) was carried out using the MethylDetector kit (Active Motive). Purified genomic DNA (1μg) was converted with bisulfite, and purified according to the manufacturer's instructions. The methylation status of the upstream transcription start site (P1 region) of the Pitx2 gene was analyzed by real-time PCR. Cycle threshold values obtained from each probe (Ctm and Ctu represent the methylated and unmethylated status, respectively) were used to calculate the methylation rate (=100/[1 + 2^{Ctm-Ctu}^]) [6]. The reverse transcription and quantitative real-time PCR were done using the ABI PRISM 7300 Sequence Detection System (Applied Biosystems). Relative quantification of gene expression was determined using the comparative CT method. Gene expression levels in A relative to B were calculated using the following formulas: ΔΔCT=ΔCTA-CTB, fold change = 2^−ΔΔCT^. Primers were shown in Supplementary Table S4.

### Immunoprecipitation and immunoblotting

Cells were lysed by sonicating for 5 s in 1 ml of detergent-free lysis buffer (PBS, 5 mM EDTA, 0.02% sodium azide, 10 mM iodoacetamide, 1 mM PMSF, and 2 mg leupeptin) at 4°C. Antibody-conjugated beads were prepared by combining 1 mg of antibodies with 30 ml of a 50% protein A Sepharose bead slurry in 0.5 ml of ice-cold PBS for 1 hr at 4°C in a tube rotator and were then washed three times with 1 ml of lysis buffer. The antibodies used for coimmunoprecipitation were Akt (ab6076, abcam). The beads were washed three times with washing buffer (50 mM Tris-HCl, 300 mM NaCl, 5 mM EDTA, 0.02% sodium azide, 0.1% Triton X-100) and once with ice-cold PBS. Immunodetection was carried out using the ECL Western Blotting Detection Kit (Amersham Corp). Proteins were detected using antibodies against Smad4 (ab40759, abcam), Pitx2 (sc-8748, Santa Cruz), p-Smad3 (ab52903, abcam), p-Smad2 (ab53100, abcam), B55α (ab185712, abcam), pAKT-T308 (ab5626, abcam), pAKT-S473 (ab8932, abcam) and GAPDH (ab9485, abcam). Relative protein expression levels were normalized to GAPDH levels.

### luciferase reporter assay and chromatin immunoprecipitation

All transfections were performed using Lipofectamine 2000 (Invitrogen). The luciferase assay was performed with the Dual Luciferase Assay System kit (Promega Corp.). Relative luciferase activity was reported as the fold induction after normalization for transfection efficiency. ChIP was performed using the EZ ChIP™ Chromatin Immunoprecipitation Kit (Millipore). Chromatin was immunoprecipitated using the mouse monoclonal anti-Smad4 antibody (ab3219, abcam) or anti-Pitx2 antibody (ab192495, abcam). Normal polyclonal rabbit anti-mouse IgG was used as negative control. Primer sequences were listed in Supplementary Table S4.

### Cell proliferation, soft agar assay and invasion assay

A detailed description was previously described [25].

### Nude mouse subcutaneous xenograft and metastasis model

The BALB/c nude mice were divided into experimental and control groups. The cells (5×10^6^ per mouse) were injected into the subcutis of nude mice in groups of five of experimental or control group, and 25 days later, tumor volume (V) was estimated using the formula V = LW^2^/6 (L: length of tumor; W: width of tumor). Also, the cells (1 ×10^6^ per mouse) were injected into the tail vein of nude mice in groups of five of experimental or control group, and 60 days later, lung of each mouse was removed and lung metastases were determined. The micrometastases in the lung tissues had been quantified by two independent pathologists (double-blinded). The BALB/c nude mice were maintained under specific pathogen-free conditions at the Shanghai Experimental Animals Centre of Chinese Academy of Sciences, and they were cared for in accordance with the institutional guidelines.

### Histology, immunohistochemistry, and immunofluorescence

Four-micrometer-thick sections were stained with hematoxylin and eosin for histological verification. Primary antibody against Pitx2 was purchased from Santa Cruz (sc-8748). To evaluate the immunohistochemistry results, the percentage of positive cells was scored from grade 0 to grade 3: Grade 0 (negative), <1% or 0% of the cells were stained; Grade 1 (weak), 1% to 49% of the cells were stained; Grade 2 (moderate), 50% to 70% of the cells were stained; and Grade 3 (intense), >70% of the cells were stained [26, 28]. For immunofluorescence staining, the antibodies used included anti-Smad4 (ab110175, abcam) and anti-Pitx2 (ab55599, abcam). Rhodamine-conjugated goat anti-rabbit IgG and FITC-conjugated goat anti-mouse IgG were used as secondary antibodies. The stained cells were mounted on glass slides and examined by confocal microscopy.

### Statistical analysis

Statistical analyses were carried out using SPSS version 17.0. Each experiment was repeated at least three times. The results were presented as mean ± SD. Significant differences were assessed using Student's *t*-test or Kruskal-Wallis test. Postoperative survival was evaluated using the Kaplan-Meier method and log-rank test. Pearson's correlation test was used for correlation analysis. *p* values less than 0.05 were considered statistically significant.

## SUPPLEMENTARY FIGURES AND TABLES





## References

[1] Han K, Chen X, Bian N, Ma B, Yang T, Cai C, Fan Q, Zhou Y, Zhao TB (2015). MicroRNA profiling identifies MiR-195 suppresses osteosarcoma cell metastasis by targeting CCND1. Oncotarget.

[2] Estevez-Garcia P, Rivera F, Molina-Pinelo S, Benavent M, Gómez J, Limón ML, Pastor MD, Martinez-Perez J, Paz-Ares L, Carnero A5, Garcia-Carbonero R (2015). Gene expression profile predictive of response to chemotherapy in metastatic colorectal cancer. Oncotarget.

[3] Kelberman D, Islam L, Holder SE, Jacques TS, Calvas P, Hennekam RC, Nischal KK, Sowden JC (2011). Digenic inheritance of mutations in FOXC1 and PITX2: correlating transcription factor function and Axenfeld-Rieger disease severity. Hum Mutat.

[4] Kioussi C, Briata P, Baek SH, Rose DW, Hamblet NS, Herman T, Ohgi KA, Lin C, Gleiberman A, Wang J, Brault V, Ruiz-Lozano P, Nguyen HD, Kemler R, Glass CK, Wynshaw-Boris A, Rosenfeld MG (2002). Identification of a Wnt/Dvl/beta-Catenin --> Pitx2 pathway mediating cell-type-specific proliferation during development. Cell.

[5] Iwata J, Tung L, Urata M, Hacia JG, Pelikan R, Suzuki A, Ramenzoni L, Chaudhry O, Parada C, Sanchez-Lara PA, Chai Y (2012). Fibroblast growth factor 9 (FGF9)-pituitary homeobox 2 (PITX2) pathway mediates transforming growth factor β (TGFβ) signaling to regulate cell proliferation in palatal mesenchyme during mouse palatogenesis. J Biol Chem.

[6] Nimmrich I, Sieuwerts AM, Meijer-van Gelder ME, Schwope I, Bolt-de Vries J, Harbeck N, Koenig T, Hartmann O, Kluth A, Dietrich D, Magdolen V, Portengen H, Look MP, Klijn JG, Lesche R, Schmitt M, Maier S, Foekens JA, Martens JW (2008). DNA hypermethylation of PITX2 is a marker of poor prognosis in untreated lymph node-negative hormone receptor-positive breast cancer patients. Breast Cancer Res Treat.

[7] Harbeck N, Nimmrich I, Hartmann A, Ross JS, Cufer T, Grützmann R, Kristiansen G, Paradiso A, Hartmann O, Margossian A, Martens J, Schwope I, Lukas A, Müller V, Milde-Langosch K, Nährig J, Foekens J, Maier S, Schmitt M, Lesche R (2008). Multicenter study using paraffin-embedded tumor tissue testing PITX2 DNA methylation as a marker for outcome prediction in tamoxifen-treated, node-negative breast cancer patients. J Clin Oncol.

[8] Weiss G, Cottrell S, Distler J, Schatz P, Kristiansen G, Ittmann M, Haefliger C, Lesche R, Hartmann A, Corman J, Wheeler T (2009). DNA methylation of the PITX2 gene promoter region is a strong independent prognostic marker of biochemical recurrence in patients with prostate cancer after radical prostatectomy. J Urol.

[9] Vinarskaja A, Schulz WA, Ingenwerth M, Hader C, Arsov C (2013). Association of PITX2 mRNA down-regulation in prostate cancer with promoter hypermethylation and poor prognosis. Urol Oncol.

[10] Hirose H, Ishii H, Mimori K, Tanaka F, Takemasa I, Mizushima T, Ikeda M, Yamamoto H, Sekimoto M, Doki Y, Mori M (2011). The significance of PITX2 overexpression in human colorectal cancer. Ann Surg Oncol.

[11] Huang Y, Guigon CJ, Fan J, Cheng SY, Zhu GZ (2010). Pituitary homeobox 2 (PITX2) promotes thyroid carcinogenesis by activation of cyclin D2. Cell Cycle.

[12] Liu Y, Huang Y, Zhu GZ (2013). Cyclin A1 is a transcriptional target of PITX2 and overexpressed in papillary thyroid carcinoma. Mol Cell Biochem.

[13] Vela I, Morrissey C, Zhang X, Chen S, Corey E, Strutton GM, Nelson CC, Nicol DL, Clements JA, Gardiner EM (2014). PITX2 and non-canonical Wnt pathway interaction in metastatic prostate cancer. Clin Exp Metastasis.

[14] Basu M, Roy SS (2013). Wnt/β-catenin pathway is regulated by PITX2 homeodomain protein and thus contributes to the proliferation of human ovarian adenocarcinoma cell, SKOV-3. J Biol Chem.

[15] Basu M, Mukhopadhyay S, Chatterjee U, Roy SS (2014). FGF16 promotes invasive behavior of SKOV-3 ovarian cancer cells through activation of mitogen-activated protein kinase (MAPK) signaling pathway. J Biol Chem.

[16] Hingorani SR, Petricoin EF, Maitra A, Rajapakse V, King C, Jacobetz MA, Ross S, Conrads TP, Veenstra TD, Hitt BA, Kawaguchi Y, Johann D, Liotta LA, Crawford HC, Putt ME, Jacks T, Wright CV, Hruban RH, Lowy AM, Tuveson DA (2003). Preinvasive and invasive ductal pancreatic cancer and its early detection in the mouse. Cancer Cell.

[17] Hingorani SR, Wang L, Multani AS, Combs C, Deramaudt TB, Hruban RH, Rustgi AK, Chang S, Tuveson DA (2005). Trp53^R172H^ and Kras^G12D^ cooperate to promote chromosomal instability and widely metastatic pancreatic ductal adenocarcinoma in mice. Cancer Cell.

[18] Song Y, Wang K, Chen DB, Magness RR, Zheng J (2009). Suppression of protein phosphatase 2 differentially modulates VEGF- and FGF2-induced signaling in ovine fetoplacental artery endothelial cells. Placenta.

[19] O'Shaughnessy RF, Welti JC, Sully K, Byrne C (2009). AKT-dependent Pp2a activity is required for epidermal barrier formation during late embryonic development. Development.

[20] Shen R, Wang Q, Cheng S, Liu T, Jiang H, Zhu J, Wu Y, Wang L (2013). The biological features of PanIN initiated from oncogenic *Kras* mutation in genetically engineered mouse models. Cancer Lett.

[21] Kuo YC, Huang KY, Yang CH, Yang YS, Lee WY, Chiang CW (2008). Regulation of phosphorylation of Thr-308 of AKT, cell proliferation, and survival by the B55alpha regulatory subunit targeting of the protein phosphatase 2A holoenzyme to AKT. J Biol Chem.

[22] Sipos B, Möser S, Kalthoff H, Török V, Löhr M, Klöppel G (2003). A comprehensive characterization of pancreatic ductal carcinoma cell lines: towards the establishment of an in vitro research platform. Virchows Arch.

[23] Deer EL, González-Hernández J, Coursen JD, Shea JE, Ngatia J, Scaife CL, Firpo MA, Mulvihill SJ (2010). Phenotype and genotype of pancreatic cancer cell lines. Pancreas.

[24] Ouyang H, Mou L, Luk C, Liu N, Karaskova J, Squire J, Tsao MS (2000). Immortal human pancreatic duct epithelial cell lines with near normal genotype and phenotype. Am J Pathol.

[25] Wang Q, Liu H, Liu T, Shu S, Jiang H, Cheng S, Yuan Y, Yang W, Wang L (2013). BRCA2 dysfunction promotes malignant transformation of pancreatic intraepithelial neoplasia. Anticancer Agents Med Chem.

[26] Wang Q, Jiang H, Ping C, Shen R, Liu T, Li J, Qian Y, Tang Y, Cheng S, Yao W, Wang L (2015). Exploring the Wnt Pathway-Associated LncRNAs and Genes Involved in Pancreatic Carcinogenesis Driven by Tp53 Mutation. Pharm Res.

[27] Wang L, Qi X, Shen R, Sun Y, Tuveson DA (2009). An shRNA silencing a non-toxic transgene reduces nutrient consumption and increases production of adenoviral vectors in a novel packaging cell. J Cell Physiol.

[28] Hotz B, Arndt M, Dullat S, Bhargava S, Buhr HJ, Hotz HG (2007). Epithelial to mesenchymal transition: expression of the regulators snail, slug, and twist in pancreatic cancer. Clin Cancer Res.

