# Supplementary material for: Smad4-dependent suppressor pituitary homeobox 2 promotes PPP2R2A-mediated inhibition of Akt pathway in pancreatic cancer
Source: Oncotarget. 2016 Feb 3;7(10):11208–22. doi: 10.18632/oncotarget.7158 (PMC4905467; doi:10.18632/oncotarget.7158)
Supplement: Supplementary file 1 [file oncotarget-07-11208-s001.pdf]

SUPPLEMENTARY FIGURES AND TABLES

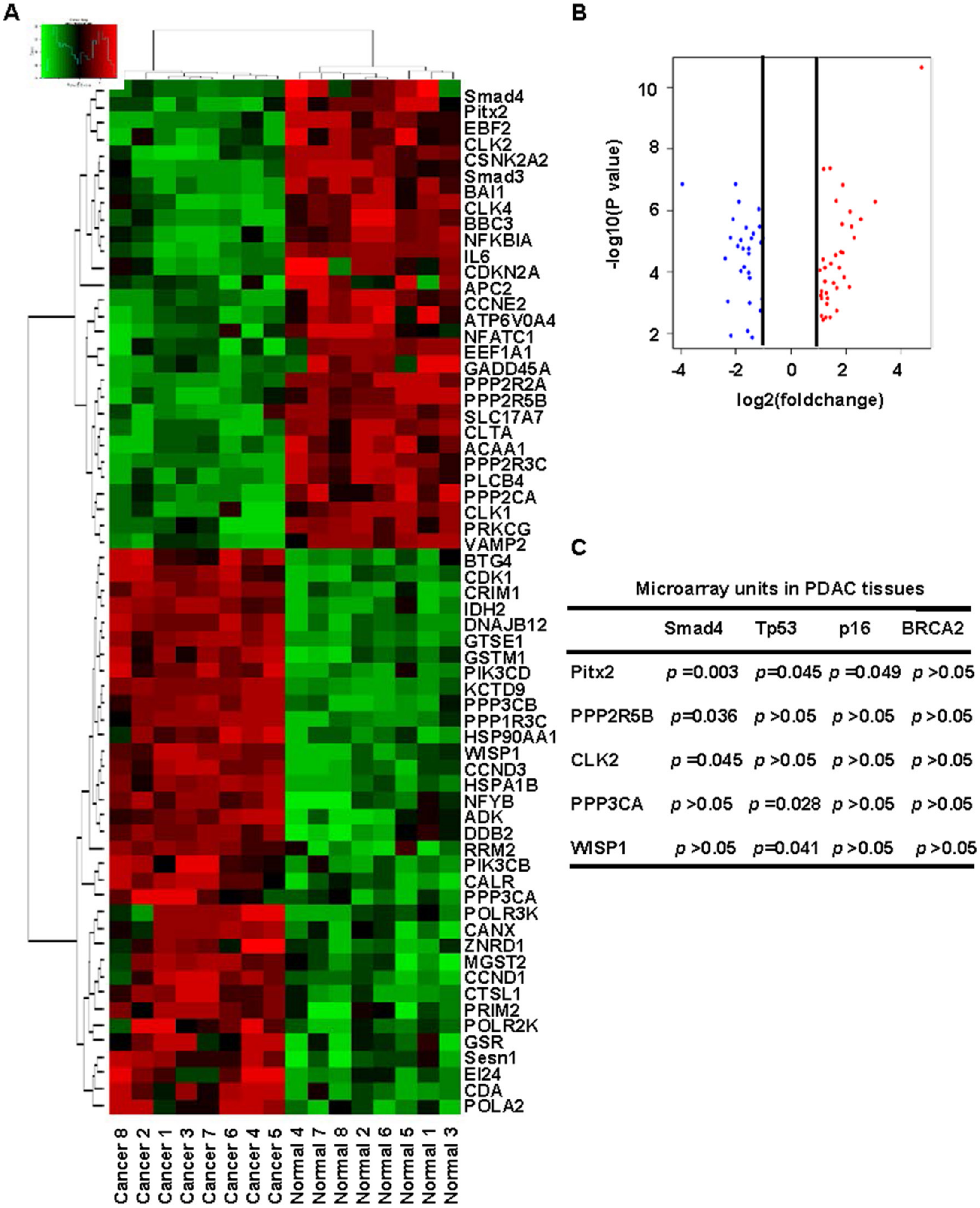

Microarray units in PDAC tissues

	Smad4	Tp53	p16	BRCA2
Pitx2	*p* =0.003	*p*=0.045	*p* =0.049	*p* >0.05
PPP2R5B	*p*=0.036	*p* >0.05	*p* >0.05	*p* >0.05
CLK2	*p* =0.045	*p* >0.05	*p* >0.05	*p* >0.05
PPP3CA	*p* >0.05	*p* =0.028	*p* >0.05	*p* >0.05
WISP1	*p* >0.05	*p*=0.041	*p* >0.05	*p* >0.05

Supplementary Figure S1: The heatmap and volcano plot filtering of the differentially expressed mRNAs in PDAC tissues compared with matched normal tissues. A. Heatmap. **B.** Volcano plot filtering. **C.** The correlation analysis between differentially expressed candidate genes and the known suppressors Smad4, Tp53, p16 or BRCA2.

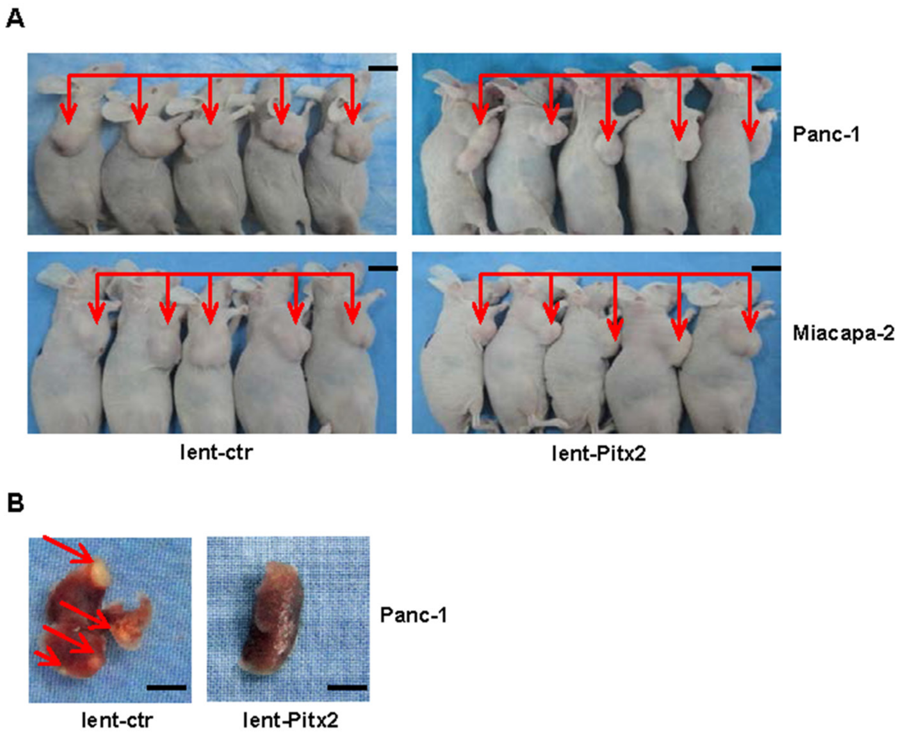

Supplementary Figure S2: The effect of Pitx2 on tumorigenicity and lung metastasis ability was investigated in PDAC cells. After lentivirus vector-intermediated Pitx2 overexpression or control (lent-Pitx2 or lent-ctr) in PDAC cells, the tumorigenicity ability **A.** and metastasis ability **B.** of cells were attenuated compared with the control(n = 5) (bar scale = 1 cm).

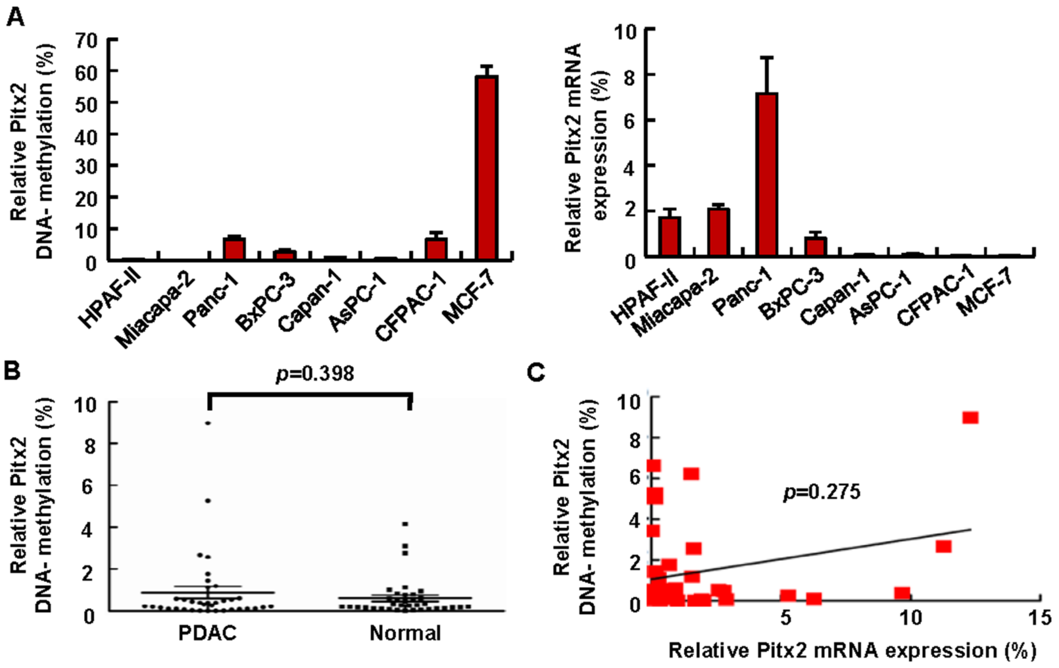

Supplementary Figure S3: Pitx2 DNA-methylation levels and mRNA levels were detected in human PDAC cell lines and human PDAC tissues. A. *Pitx2* DNA-methylation levels and *Pitx2* mRNA levels were detected by methylation-specific PCR and real-time PCR in human PDAC cell lines. Human breast cancer cell line MCF-7 with high *Pitx2* DNA-methylation was used as positive control. **B.** Methylation-specific PCR analysis showed low *Pitx2* DNA methylation levels in 36 fresh human PDAC tissues and matched paraneoplastic pancreas tissues (right panel). Pearson correlation test showed no correlation between *Pitx2* DNA-methylation levels and *Pitx2* mRNA levels in 36 human PDAC tissues (left panel).

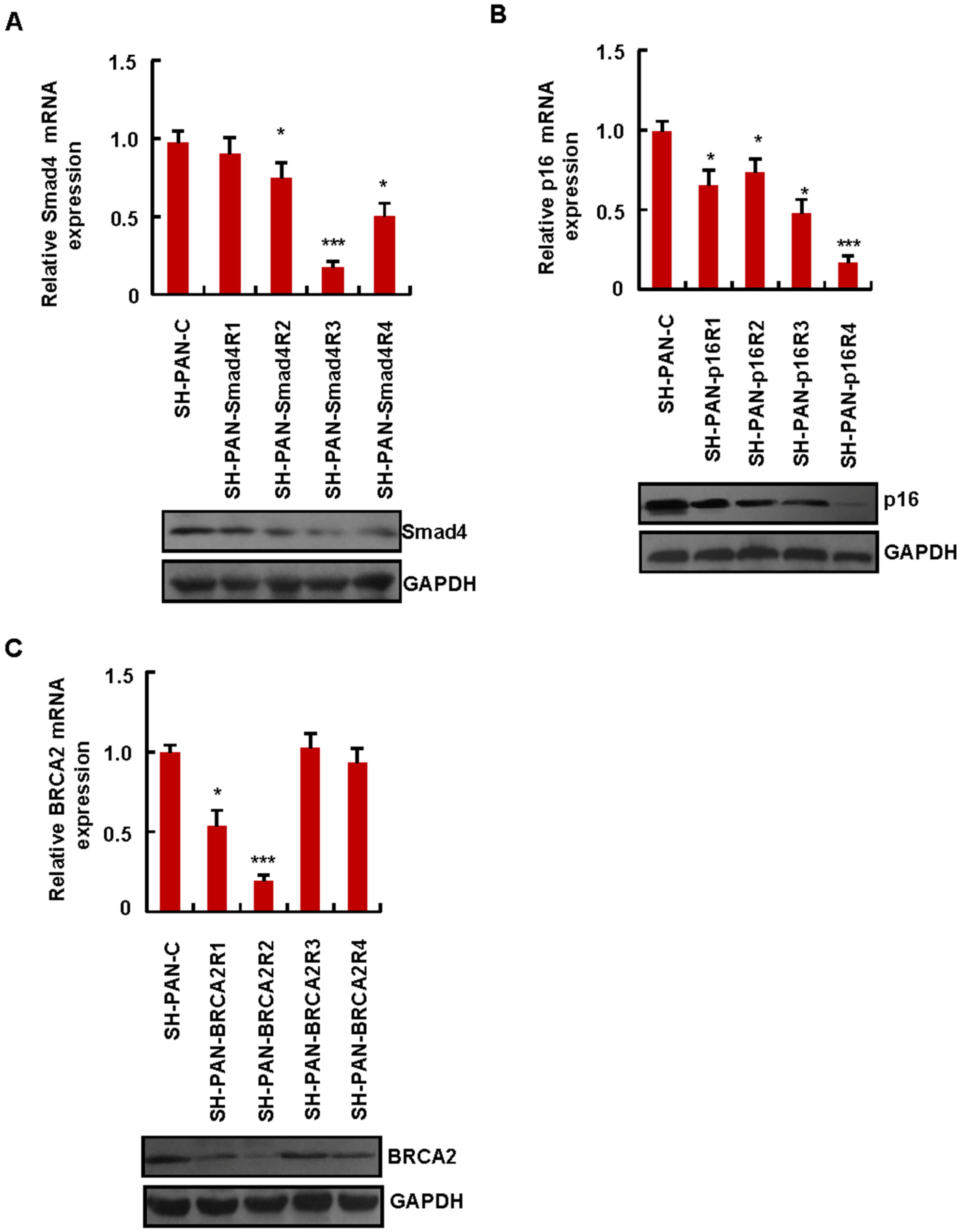

Supplementary Figure S4: The knockdown effect of Smad4, p16 and BRCA2 in SH-PAN cell line. Real-time PCR and Western blot analysis were performed to show the knockdown effect of Smad4 **A.** p16 **B.** and BRCA2 **C.** in SH-PAN cell line (*, $P < 0.05$; ***, $p < 0.001$).

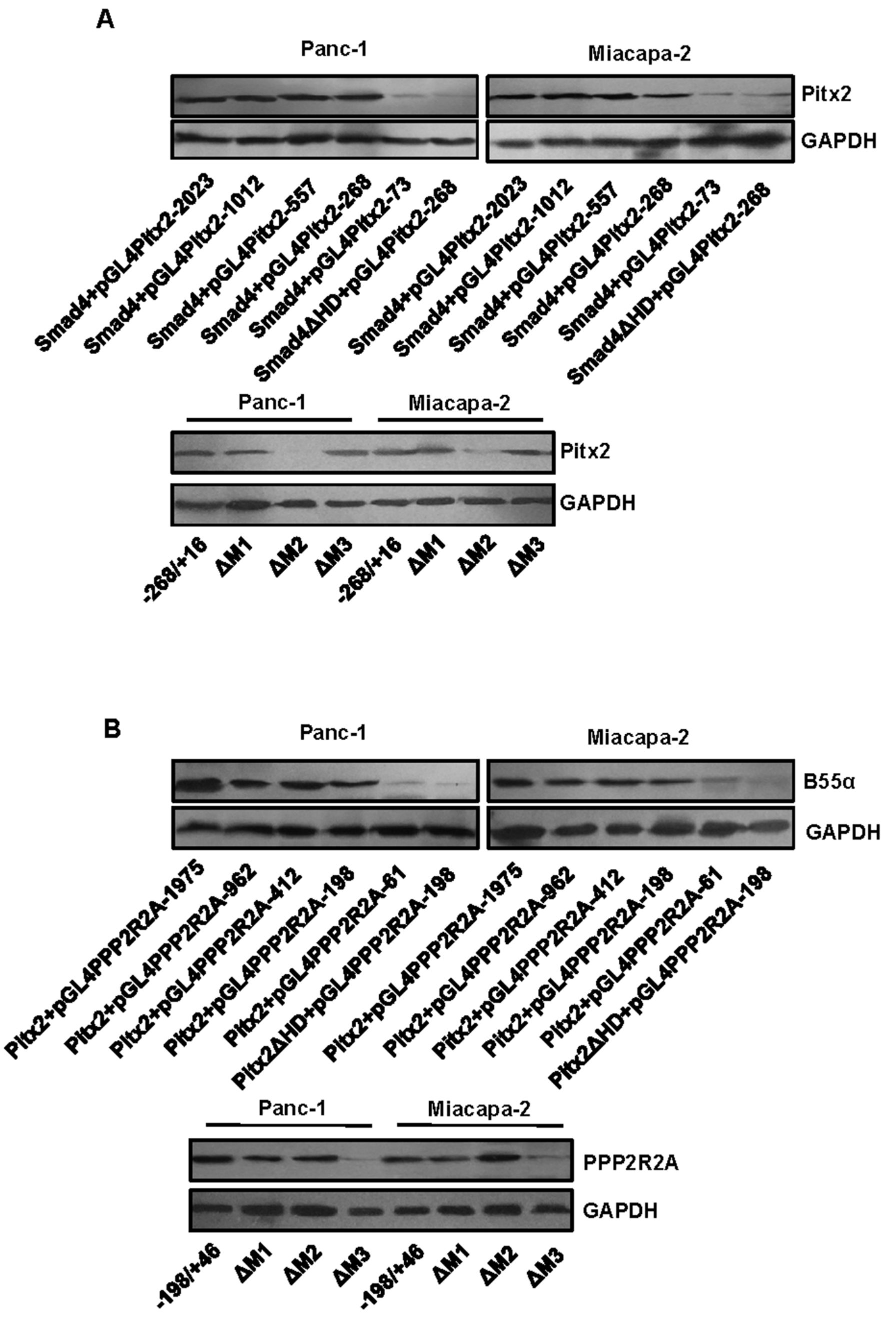

Supplementary Figure S5: The protein levels of Pitx2 and PPP2R2A in human PDAC cell lines. Western blot analysis were performed to show the protein levels of Pitx2 **A.** and PPP2R2A **B.** in the luciferase promoter–reporter assays in Panc-1 and Miacapa-2 cells.

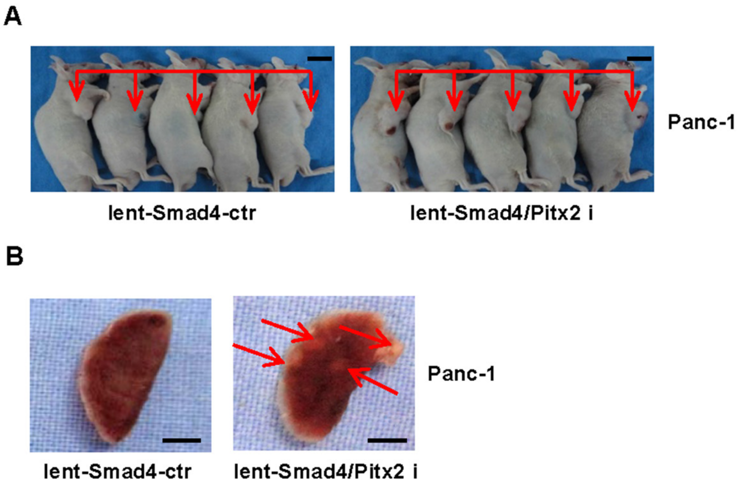

Supplementary Figure S6: Knockdown of Pitx2 enhanced the tumorigenicity and micrometastasis of Panc-1 cells with Smad4-overexpression. Lentiviral vector-intermediated *Smad4*- overexpression/*Pitx2*-shRNA (namely Panc-1-lent-Smad4/Pitx2i) or lentiviral vector- intermediated *Smad4*-overexpression/ control-shRNA (namely Panc-1-lent-Smad4/ctr) were stably cotransduced into Panc-1 cells, respectively. The subcutaneous tumorigenicity ability **A.** and lung metastasis ability post tail vein injection of Panc-1 cells **B.** were compared. Knockdown of *Pitx2* enhanced the tumorigenicity and micrometastasis ability in Panc-1 cells with Smad4-overexpression.

Supplementary Table S1: The differentially expressed mRNAs in human PDAC tissues compared to paraneoplastic tissues

Seqname	GeneSymbol	p-value	FC Absolute	Regulation
NM_001786.4	CDK1	8.96E-05	2.073291	up
NM_053056.2	CCND1	7.59E-05	3.385678	up
NM_004343.3	CALR	2.95E-05	3.077135	up
NM_001746.3	CANX	0.000233	2.927082	up
NM_001912.4	CTSL1	2.22E-05	3.492202	up
NM_000107.2	DDB2	0.001075	2.463901	up
NM_001785.2	CDA	0.000336	3.159275	up
NM_000637.3	GSR	0.002655	2.183998	up
NM_006219.2	PIK3CB	0.000145	3.852775	up
NM_000944.4	PPP3CA	0.001815	3.162072	up
NM_001136017.3	CCND3	4.89E-07	3.133273	up
NM_000561.3	GSTM1	2.35E-05	3.701566	up
NM_004879.4	EI24	0.003038	2.689423	up
NM_002689.3	POLA2	0.000478	2.425326	up
NM_001142353.2	PPP3CB	3.48E-06	4.588684	up
NM_014454.2	Sesn1	0.000419	2.176808	up
NM_005034	POLR2K	0.000711	2.542403	up
NM_002168	IDH2	2.89E-06	3.635826	up
NM_005026	PIK3CD	7.67E-06	4.892173	up
NM_016426	GTSE1	4.20E-08	2.705906	up

(*Continued*)

Seqname	GeneSymbol	p-value	FC Absolute	Regulation
NM_017589.3	BTG4	5.35E-05	2.722477	up
NM_001034	RRM2	0.000197	2.344561	up
NM_005348	HSP90AA1	3.91E-05	2.228297	up
NM_016310	POLR3K	0.003611	2.234887	up
NM_000947	PRIM2	0.0007	2.176687	up
NM_005346	HSPA1B	4.51E-08	2.292881	up
NM_006166	NFYB	7.41E-05	2.376705	up
NM_014596	ZNRD1	0.003005	2.416298	up
NM_002413	MGST2	0.0006	2.143559	up
NM_017634	KCTD9	2.29E-11	26.79315	up
NM_001123	ADK	0.000322	4.373881	up
NM_003882	WISP1	1.98E-06	5.808422	up
NM_005398	PPP1R3C	5.27E-07	8.305961	up
NM_016441	CRIM1	1.09E-06	4.442047	up
NM_017626	DNAJB12	1.43E-07	3.685148	up
NM_001607	ACAA1	1.14E-05	2.131691	down
NM_001702	BAI1	3.57E-06	3.076192	down
NM_006162	NFATC1	0.00075	2.052235	down
NM_130840	ATP6V0A4	0.000157	2.857907	down
NM_000325	Pitx2	3.68E-05	5.228792	down
NM_006244	PPP2R5B	1.89E-06	4.296272	down
NM_001076677	CLTA	9.34E-07	2.256585	down
NM_014417	BBC3	0.000109	2.864635	down
NM_002717	PPP2R2A	7.57E-06	4.580571	down
NM_001145104	Smad3	3.34E-06	2.225007	down
NM_001924	GADD45A	0.001835	2.171643	down
NM_003993	CLK2	0.000917	4.906759	down
NM_020309	SLC17A7	2.59E-05	2.880993	down
NM_022659	EBF2	9.30E-06	3.514082	down
NM_004071	CLK1	6.86E-05	3.212469	down
NM_020666	CLK4	1.43E-05	3.811688	down
NM_000600	IL6	1.34E-07	15.51283	down
NM_000077.4	CDKN2A	0.008043	2.974181	down
NM_014232	VAMP2	8.43E-06	2.027226	down
NM_001402	EEF1A1	9.67E-05	3.548814	down
NM_020529	NFKBIA	1.78E-05	3.330405	down
NM_001172646	PLCB4	5.31E-07	3.687952	down

(*Continued*)

Seqname	GeneSymbol	p-value	FC Absolute	Regulation
NM_001896	CSNK2A2	5.47E-06	2.555645	down
NM_005359	Smad4	0.012261	4.549878	down
NM_005883	APC2	0.01371	2.63851	down
NM_002715	PPP2CA	1.77E-05	2.902772	down
NM_002739	PRKCG	0.001035	2.84366	down
NM_057749	CCNE2	8.27E-06	2.688858	down
NM_017917	PPP2R3C	1.34E-07	3.992405	down

Note: The differentially expressed mRNAs changes (≥2-fold and *p*-value< 0.05) were listed.

Supplementary Table S2: The differentially expressed mRNAs in HPDE6-C7-lent-Pitx2i cells compared to HPDE6-C7-lent-ctr cells

Seqname	GeneSymbol	p-value	FC Absolute	Regulation
NM_012116	CBLC	1.77E-08	27.86552	up
NM_001172430	AMPD3	0.005205	27.24157	up
NM_006430	CCT4	0.002134	11.06076	up
NM_175735	LYG2	0.001032	9.748033	up
NM_001136473	LITAF	0.000934	7.591369	up
NM_001007243	STAR	6.24E-07	7.39777	up
NM_014357	LCE2B	0.000923	6.570721	up
NM_002880	RAF1	0.025882	6.404283	up
NM_016486	TMEM69	0.000586	6.398624	up
NM_021824	NIF3L1	4.14E-05	6.160948	up
NM_133274	FCAR	0.009203	5.599027	up
NM_005102	FEZ2	0.015199	5.24231	up
NM_015146	41525	0.009832	5.241029	up
NM_006943	SOX12	0.007983	4.724354	up
NM_016029	DHRS7	0.026563	4.559863	up
NM_015143	METAP1	0.005491	4.504227	up
NM_001321	CSRP2	0.002337	4.263428	up
NM_006230	POLD2	0.002034	4.254721	up
NM_148919	PSMB8	0.000155	4.031554	up
NM_005917	MDH1	0.005641	3.961092	up
NM_001223	CASP1	8.83E-05	3.863232	up
NM_005697	SCAMP2	0.047882	3.767864	up
NM_001130015	ANKRD33	0.003142	3.489638	up
NM_021960	MCL1	0.021028	3.458298	up

(*Continued*)

Seqname	GeneSymbol	p-value	FC Absolute	Regulation
NM_001103148	GIGYF2	0.004508	3.335373	up
NM_001949	E2F3	0.024219	3.286434	up
NM_182524	ZNF595	0.012098	3.146712	up
NM_001142641	FBRSL1	0.010783	3.063235	up
NM_007354	C3orf27	0.002407	2.94881	up
NM_138476	MDP1	0.018868	2.924877	up
NM_001099696	KIAA0101	0.019662	2.914865	up
NM_182976	ZNF326	0.009942	2.910392	up
NM_001170	Aqp7	0.002217	2.909244	up
NM_017836	SLC41A3	0.009766	2.892016	up
NM_176787	PIGN	0.005013	2.873419	up
NM_205854	SFTA2	0.003421	2.869733	up
NM_133179	XAGE3	0.004092	2.849057	up
NM_003028	SHB	0.045962	2.847109	up
NM_014858	TMCC2	0.028664	2.785642	up
NM_012108	STAP1	0.046014	2.74565	up
NM_001080517	SETD5	0.038012	2.699317	up
NM_003768	PEA15	0.005361	2.689365	up
NM_001295.2	Ccr1	0.002332	2.640108	up
NM_001010922	BCL2L15	0.005135	2.61698	up
NM_018890	Rac1	0.0003	2.592211	up
NM_000944.4	PPP3ca	0.0009	2.586112	up
NM_152357	ZNF440	0.001347	2.490001	up
NM_005730	CTDSP2	1.27E-05	2.457112	up
NM_000972	Rpl7a	0.0009	2.445992	up
NM_016548.3	Golm1	3.43E-05	2.439228	up
NM_012423	RPL13A	0.009332	2.350027	up
NM_001080400	PLIN4	4.97E-06	2.340247	up
NM_138791	C14orf148	0.012849	2.293019	up
NM_005621	S100A12	2.81E-05	2.275558	up
NM_001163523	ITPRIPL1	0.001783	2.271357	up
NM_001097589	SPRR3	0.007079	2.232479	up
NM_003583	DYRK2	0.001573	2.224143	up
NM_182981	OSGIN1	0.010095	2.209631	up
NM_001085372	C11orf83	0.011127	2.180972	up
NM_001029835	CCM2	0.043593	2.178263	up
NM_144647	CAPSL	0.000182	2.170438	up

(*Continued*)

Seqname	GeneSymbol	p-value	FC Absolute	Regulation
NM_006098	GNB2L1	0.022941	2.113265	up
NM_144687	NLRP12	0.004017	2.111171	up
NM_152406	AFAP1L1	0.010413	2.100748	up
NM_001001715	FARP1	3.14E-06	2.094003	up
NM_144596	TTC8	0.006007	2.090919	up
NM_213589	RAPH1	1.94E-06	2.078049	up
NM_144577	CCDC114	0.02203	2.054021	up
NM_001024957	BRMS1	0.000277	2.041239	up
NM_182705	FAM101B	0.011929	2.011674	up
NM_178273	PILRA	0.024843	2.007355	up
NM_138464	C5orf55	0.007941	2.002969	down
NM_001130915	MAMSTR	0.013262	2.02837	down
NM_001170760	SRPK3	0.008219	2.031436	down
NM_021138	TRAF2	0.000116	2.076423	down
NM_013230	CD24	0.043072	2.089968	down
NM_001632	ALPP	0.005807	2.092023	down
NM_012091	ADAT1	0.007897	2.126445	down
NM_005028	PIP4K2A	0.030777	2.171448	down
NM_001109903	RNFT2	0.00554	2.17455	down
NM_004860	FXR2	0.001447	2.243502	down
NM_017806	LIME1	0.00213	2.255228	down
NM_003373	VCL	0.0017	2.268378	down
NM_014736	REPIN1	0.002838	2.274559	down
NM_207418	FAM72D	0.015903	2.29949	down
NM_178351.3	Lce1c	0.003532	2.309795	down
NM_001001998	EXOSC10	0.001166	2.330596	down
NM_182720	CREM	0.001221	2.342274	down
NM_003753	EIF3D	0.021008	2.35581	down
NM_181291	WDR20	0.000384	2.402932	down
NM_001127235	Gpbp1	0.003601	2.422176	down
NM_017917	PPP2R3C	0.0015	2.427998	down
NM_030984	Tbxas1	0.0206	2.452182	down
NM_001007229	SPOP	0.005253	2.459922	down
NM_012383	OSTF1	0.000526	2.515793	down
NM_001759.3	CCND2	0.0003	2.577306	down
NM_152836	SNX16	3.21E-06	2.651403	down
NM_016604	KDM3B	0.010547	2.657719	down

(*Continued*)

Seqname	GeneSymbol	p-value	FC Absolute	Regulation
NM_014813	LRIG2	0.033189	2.727812	down
NM_015844	MBD1	0.030873	2.786575	down
NM_133635	POFUT2	0.000596	2.798057	down
NM_016009	SH3GLB1	0.000108	2.906689	down
NM_006172	Nppa	0.020014	2.910104	down
NM_031892	SH3KBP1	0.002736	2.954174	down
NM_001130012	SLC9A3R2	0.000424	3.120808	down
NM_005713	COL4A3BP	0.005455	3.280197	down
NM_001099653	FAM86C	0.000457	3.291593	down
NM_012352	OR1A2	0.03204	3.505878	down
NM_015939	TRMT6	0.007113	3.735301	down
NM_001146003	NEK11	6.39E-05	3.800804	down
NM_018113	LMBR1L	0.000267	3.808804	down
NM_177478	FTMT	0.03471	3.896101	down
NM_181077	GOLGA8A	4.16E-06	4.070191	down
NM_002717	PPP2R2A	0.002	4.113653	down
NM_001146695	KDM4C	0.002788	4.263817	down
NM_178865	SERINC2	7.35E-05	4.769531	down
NM_020682	AS3MT	0.011538	4.950763	down
NM_001137664	ANAPC7	4.32E-05	5.540234	down
NM_001164267	WDR46	4.14E-05	5.714443	down
NM_181614	KRTAP19-7	0.014029	6.615148	down
NM_001178086	ZFX	2.16E-05	7.375199	down
NM_006923	SDF2	0.003177	7.674646	down
NM_001098407	GAGE2D	0.004909	8.065318	down
NM_001142762	C15orf23	0.000138	11.09798	down
NM_001170416	TJP2	0.01202	14.06263	down
NM_000995	RPL34	0.000543	20.87205	down
NM_002775	HTRA1	0.009126	42.62645	down
NM_018896	CACNA1G	0.001253	131.1168	down

Note: The differentially expressed mRNAs changes (≥2-fold and *p*-value< 0.05) were listed.

Supplementary Table S3: The differentially expressed mRNAs in Panc-1-lent-Pitx2 cells compared to Panc-1-lent-ctr cells

Seqname	GeneSymbol	p-value	FC Absolute	Regulation
NM_003435	ZNF134	0.00222	17.22466	up
NM_002717	PPP2R2A	3.01E-06	9.839773	up
NM_002766	PRPSAP1	0.000242	9.42873	up
NM_057158	DUSP4	0.001407	7.840217	up
NM_005984	SLC25A1	0.000189	7.585614	up
NM_001729	BTC	0.006782	7.102346	up
NM_003792	EDF1	8.75E-05	4.869893	up
NM_172070	UBR3	0.006628	4.789471	up
NM_017443	POLE3	0.000519	4.502314	up
NM_015401	HDAC7	0.000995	4.324728	up
NM_001167868	ATP5SL	0.040217	4.214165	up
NM_006172	Nppa	1.44E-05	4.031553	up
NM_016558	SCAND1	0.000471	3.949286	up
NM_001100590	KIAA0232	0.017488	3.664999	up
NM_001759.3	CCND2	0.0004	3.611718	up
NM_002107	H3F3A	0.002708	3.60408	up
NM_001127235	Gpbp1	0.0013	3.397419	up
NM_206825	GNL3	0.000146	3.356782	up
NM_001080468	SYCN	0.002786	3.242504	up
NM_017917	PPP2R3C	0.0005	3.137749	up
NM_001040280	CD83	4.57E-05	3.103446	up
NM_001134388	ZDHHC4	2.59E-05	3.090736	up
NM_025075	THOC7	0.005932	3.010424	up
NM_005700	DPP3	0.005318	3.002126	up
NM_174926	TMEM136	7.35E-06	2.785766	up
NM_005762	TRIM28	0.029665	2.778139	up
NM_016828	OGG1	0.003747	2.716146	up
NM_199280	FAM179A	0.010377	2.700614	up
NM_033000	GTF2I	0.0187	2.684908	up
NM_001207	BTF3	0.031154	2.676397	up
NM_030984	Tbxas1	0.0005	2.576355	up
NM_003978	PSTPIP1	0.032896	2.562826	up
NM_001174069	LMX1A	0.000503	2.556352	up
NM_000941	POR	0.019582	2.55605	up
NM_001020	RPS16	0.023168	2.530886	up
NM_001142432	MORF4L2	0.00866	2.51452	up

(*Continued*)

Seqname	GeneSymbol	p-value	FC Absolute	Regulation
NM_006440	TXNRD2	0.04436	2.510958	up
NM_001515	GTF2H2	0.000315	2.410347	up
NM_001126242	TP73	0.000208	2.410081	up
NM_198881	TBC1D8B	0.002535	2.366561	up
NM_001143966	TBC1D7	0.00396	2.349344	up
NM_001135822	FDPS	0.004441	2.320498	up
NM_004282	BAG2	0.000659	2.315384	up
NM_018904	PCDHA13	0.017432	2.301471	up
NM_002408	MGAT2	0.001623	2.284173	up
NM_173087	CAPN3	0.000197	2.267808	up
NM_001024463	PPIP5K1	0.0096	2.251166	up
NM_178351.3	Lce1c	0.0048	2.221976	up
NM_013241	FHOD1	0.018505	2.154452	up
NM_182508	C13orf30	0.035185	2.134802	up
NM_002629	PGAM1	0.015289	2.130092	up
NM_001135740	PNCK	0.006761	2.111423	up
NM_003055	SLC18A3	0.005964	2.107611	up
NM_007101	SARDH	5.58E-05	2.097041	up
NM_001097616	GPR89C	0.001475	2.08806	up
NM_022835	PLEKHG2	0.031915	2.059897	up
NM_172160	KCNAB1	0.042335	2.051117	up
NM_138452	DHRS1	0.004888	2.017775	up
NM_003782	B3GALT4	2.82E-05	2.010348	down
NM_001128850	RRAD	0.046696	2.020506	down
NM_006683	EDDM3A	0.000146	2.026392	down
NM_015322	FEM1B	0.011791	2.02675	down
NM_031219	HDHD3	0.006376	2.079253	down
NM_001025070	RPS14	0.004483	2.159614	down
NM_018982	YIPF1	0.017166	2.199664	down
NM_001241	CCNT2	0.003594	2.217111	down
NM_016548.3	Golm1	0.0001	2.272991	down
NM_001163286	EWSR1	0.001387	2.2939	down
NM_181893	UBE2D3	0.002815	2.330061	down
NM_001134363	RBM20	0.009797	2.407311	down
NM_002172	IFNA14	0.0407	2.437774	down
NM_017654	SAMD9	0.034184	2.460556	down
NM_152391	PQLC3	0.001172	2.462377	down

(*Continued*)

Seqname	GeneSymbol	p-value	FC Absolute	Regulation
NM_001170	Aqp7	0.0027	2.542336	down
NM_000972	Rpl7a	2.15E-05	2.597005	down
NM_005297	MCHR1	0.031688	2.639638	down
NM_007238	PXMP4	0.002142	2.735387	down
NM_000944.4	PPP3ca	1.24E-05	2.784996	down
NM_001295.2	Ccr1	0.0112	2.867088	down
NM_005567	LGALS3BP	3.72E-06	2.87761	down
NM_015140	TTLL12	0.010392	2.919998	down
NM_017452	STAU1	0.044633	2.934971	down
NM_001080425	BEX4	0.002507	2.961732	down
NM_001536	PRMT1	0.016337	3.043974	down
NM_018890	Rac1	0.0006	3.044149	down
NM_005670	EPM2A	0.000764	3.305871	down
NM_014949	KIAA0907	0.002283	3.31988	down
NM_001142749	KIAA1324L	0.00042	3.332387	down
NM_181809	BMP8A	0.002641	3.9847	down
NM_017699	SIDT1	0.000302	4.081566	down
NM_058180	C21orf58	0.000221	4.610795	down
NM_133447	AGAP11	1.31E-05	5.231667	down
NM_000915	OXT	0.004195	5.771941	down
NM_003864	SAP30	1.91E-07	6.49011	down
NM_152882	PTK7	1.31E-05	6.779881	down
NM_178019	CATSPER3	0.001623	7.908825	down
NM_001048230	ADORA1	0.000351	9.323494	down
NM_153007	ODF4	0.015379	9.886684	down
NM_001135195	SLC39A5	0.002139	10.02683	down
NM_178548	TFAP2E	4.37E-05	10.37294	down
NM_003703	NOP14	0.000111	11.16931	down
NM_001037442	RUFY3	1.22E-06	13.59972	down
NM_001077261	NCOR2	0.001229	14.88325	down
NM_000404	GLB1	2.43E-07	16.6659	down
NM_031212	SLC25A28	1.34E-05	22.97896	down
NM_194326	RPS19BP1	0.000376	33.71211	down
NM_001114726	PRRT4	0.001483	106.6829	down

Note: The differentially expressed mRNAs changes (≥2-fold and *p*-value< 0.05) were listed.

Supplementary Table S4: Primers used in this study

See Supplementary File 1
