# Supplementary material for: Smad4-dependent suppressor pituitary homeobox 2 promotes PPP2R2A-mediated inhibition of Akt pathway in pancreatic cancer
Source: Oncotarget. 2016 Feb 3;7(10):11208–22. doi: 10.18632/oncotarget.7158 (PMC4905467; doi:10.18632/oncotarget.7158)
Supplement: Supplementary file 2 [file oncotarget-07-11208-s002.docx]

**Supplementary Table S4. Primers used in this study**

| **Name** | | **Sequence (5’-3’)** | | **Product size** |
| --- | --- | --- | --- | --- |
| **Real-time PCR** | | | | |
| Pitx2 | F: ACTTTCCAGAGGAACCGCTAC | | | 233 |
| (human) | R: GCCCAGTTGTTGTAGGAATAGC | | |  |
| Smad4 | F: CCCAGGATCAGTAGGTGGAATAG | | | 245 |
| (human) | R: AGTCTAAAGGTTGTGGGTCTGC | | |  |
| Tp53 | F: catttgcacctacctcacagag | | | 197 |
| (human) | R: cagagacttgacaactccctct | | |  |
| CDKN2A | F: ctcagacatccccgattgaaag | | | 209 |
| (human) | R: atggacatttacggtagtgggg | | |  |
| BRCA2 | F: ctcagcgtttgtgtatcggg | | | 178 |
| (human) | R: ctcccacctcagcttctcaa | | |  |
| CDKN2A | F: Agagctaaatccggcctcag | | | 190 |
| (mouse) | R: ctcatgccattcctttcctgtc | | |  |
| Smad4 | F: GTCCTTACCCACTGAAGGACAT | | | 224 |
| (mouse) | R: ACCAGTACTCAGGAGCAGGATG | | |  |
| BRCA2  (mouse) | F: CTGCTGACTCTCCCGCTACTTTG  R: TTCTTGCTGGTTTTTGTTTTCACTG | | | 116  171 |
| Pitx2 | F: CCTTGGACTCCTCCAAACATAG | | |  |
| (mouse) | R: GCTGGCTAGTGAAATGAGTCCT | | |  |
| PPP3CA | F: TCTTATGAAGGAGGGAAGGCTG | | | 226 |
| (human) | R: GTACCCTCTGTCAACATAGTCCC | | |  |
| Rpl7a | F: CAGACCAGAGACAAAGCAAGAG | | | 154 |
| (human) | R:CTGAGCTTTCTTGTTCTCCACC | | |  |
| CCR1 | F: GGTTTTATGGGTCAGAGTTCCG | | | 194 |
| (human) | R:CTTTGAGTTAAGGGCCTTCTCG | | |  |
| Rac1 | F: GACACGATCGAGAAACTGAAGG | | | 225 |
| (human) | R:GAGGGGCTGAGACATTTACAAC | | |  |
| AQP7 | F: GAGCTACCTTGGTGTCAACTTG | | | 216 |
| (human) | R: GCCGTGTAGAAGAGACTGTAGA | | |  |
| GOLM1 | F:CAAGACCAGTTAAAGACCCTGC | | | 171 |
| (human) | R: GACCTCTTCTATTCGCTCCTCA | | |  |
| NPPA | F: CCTTTAGAAGATGAGGTCGTGC | | | 182 |
| (human) | R: TTTAGGAGGGCAGATCGATCAG | | |  |
| PPP2R2A | F: GAGCTGGAGGAGGGAATGATAT | | | 173 |
| (human) | R: CTCCTGCTCCTGTTGAAAGATG | | |  |
| Lce1c | F: CTAGAAGAGCAGAATCCAGGACC | | | 152 |
| (human) | R:CAGGAGAGGATCTGAGTTTCTGG | | |  |
| CCND2 | F:CTGAATACTCCTCCCCTCTTCTC | | | 168 |
| (human) | R:GTCTGAGGAATGTTGTGATGGG | | |  |
| PPP2R3C | F:CCCGGTTTTATTATAGGCTGCC | | | 175 |
| (human) | R: TCCTCTCCAATCATAGGTGGTG | | |  |
| **Name** | **Sequence (5’-3’)** | | | **Product size** |
| GPBP1 | F: CGCTCAAATTCCTCTTCTCCTG | | | 174 |
| (human) | R: TGAGTCGTCATCCTTCTCTGAG | | |  |
| TBXAS1 | F: CCTCTGTGTGGGTACTATCTTGG | | | 185 |
| (human) | R: CCTCTGACCTCTTCCCATCTTT | | |  |
| GAPDH | F: CAAAAGGGTCATCATCTCTGCC | | | 179 |
| (human) | R: TCATGAGTCCTTCCACGATACC | | |  |
| GAPDH | F: GGAGTAAGAAACCCTGGACCA | | | 163 |
| (mouse) | R: GGTATTCAAGAGAGTAGGGAGGG | | |  |
|  |  | | |  |
| **BRCA2 shRNA (mouse)** | | | |  |
| SR-1F | TGCGTAGAACCAGAAATCTACATTCAAGAGATGTAGATTTCTGGTTCTACGCTTTTTTC | | |  |
| SR-1R | TCGAGAAAAAAGCGTAGAACCAGAAATCTACATCTCTTGAATGTAGATTTCTGGTTCTACGCA | | |  |
| SR-2F | TGCAGGAGATCGGCTCTAAAGATTCAAGAGATCTTTAGAGCCGATCTCCTGCTTTTTTC | | |  |
| SR-2R | TCGAGAAAAAAGCAGGAGATCGGCTCTAAAGATCTCTTGAATCTTTAGAGCCGATCTCCTGCA | | |  |
| SR-3F | TGCAGCCCAAGTGTTGATTACATTCAAGAGATGTAATCAACACTTGGGCTGCTTTTTTC | | |  |
| SR-3R | TCGAGAAAAAAGCAGCCCAAGTGTTGATTACATCTCTTGAATGTAATCAACACTTGGGCTGCA | | |  |
| SR-4F | TGGTACTGGCTCTATTACTTCATTCAAGAGATGAAGTAATAGAGCCAGTACCTTTTTTC | | |  |
| SR-4R | TCGAGAAAAAAGGTACTGGCTCTATTACTTCATCTCTTGAATGAAGTAATAGAGCCAGTACCA | | |  |
| NC-F | GATCCTTCTCCGAACGTGTCACTTCAAGAGAGTGACACGTTCGGAGAATTTTTTG | | |  |
| NC-R | AATTCAAAAAATTCTCCGAACGTGTCACTCTCTTGAATGACACGTTCGGAGAAG | | |  |
| **Smad4 shRNA (mouse)** | | | | |
| SR-1F | TGCTGTGAGAATGCACAATCGCCGGAGTTTTGGCCACTGACTGACTCCGGCGAGTGCATTCTCA | | |  |
| SR-1R | CCTGTGAGAATGCACTCGCCGGAGTCAGTCAGTGGCCAAAACTCCGGCGATTGTGCATTCTCAC | | |  |
| SR-2F | TGCTGTTTACATTCCAACTGCACTCCGTTTTGGCCACTGACTGACGGAGTGCATGGAATGTAAA | | |  |
| SR-2R | CCTGTTTACATTCCATGCACTCCGTCAGTCAGTGGCCAAAACGGAGTGCAGTTGGAATGTAAAC | | |  |
| SR-3F | TGCTGAATGCAAGCTCATTGTGAACTGTTTTGGCCACTGACTGACAGTTCACAGAGCTTGCATT | | |  |
| SR-3R | CCTGAATGCAAGCTCTGTGAACTGTCAGTCAGTGGCCAAAACAGTTCACAATGAGCTTGCATTC | | |  |
| **Name** | **Sequence (5’-3’)** | | | **Product size** |
| SR-4F | TGCTGTGGTGAGGCAAATTAGGTGTGGTTTTGGCCACTGACTGACCACACCTATTGCCTCACCA | | |  |
| SR-4R | CCTGTGGTGAGGCAATAGGTGTGGTCAGTCAGTGGCCAAAACCACACCTAATTTGCCTCACCAC | | |  |
| NC-F | GATCCTTCTCCGAACGTGTCACTTCAAGAGAGTGACACGTTCGGAGAATTTTTTG | | |  |
| NC-R | AATTCAAAAAATTCTCCGAACGTGTCACTCTCTTGAATGACACGTTCGGAGAAG | | |  |
| **Smad4 shRNA (human)** | | | | |
| hSR-1F | GATCCGCCAGCTACTTACCATCATAATTCAAGAGATTATGATGGTAAGTAGCTGGCTTTTTTG | | |  |
| hSR-1R | AATTCAAAAAAGCCAGCTACTTACCATCATAATCTCTTGAATTATGATGGTAAGTAGCTGGCG | | |  |
| hSR-2F | GATCCGGTGTTCCATTGCTTACTTTGTTCAAGAGACAAAGTAAGCAATGGAACACCTTTTTTG | | |  |
| hSR-2R | AATTCAAAAAAGGTGTTCCATTGCTTACTTTGTCTCTTGAACAAAGTAAGCAATGGAACACCG | | |  |
| hSR-3F | GATCCGCTGGATTGAAATTCACTTACTTCAAGAGAGTAAGTGAATTTCAATCCAGCTTTTTTG | | |  |
| hSR-3R | AATTCAAAAAAGCTGGATTGAAATTCACTTACTCTCTTGAAGTAAGTGAATTTCAATCCAGCG | | |  |
| hSR-4F | GATCCGGTGGAGAGAGTGAAACATTTCAAGAGAATGTTTCACTCTCTCCACCTTTTTTG | | |  |
| hSR-4R | AATTCAAAAAAGGTGGAGAGAGTGAAACATTCTCTTGAAATGTTTCACTCTCTCCACCG | | |  |
| hNC-F | GATCCTTGCGCAACTGTGTCACGTTTCAAGAGAACGTGACACAGTTGCGCAATTTTTTG | | |  |
| hNC-R | AATTCAAAAAATTGCGCAACTGTGTCACGTTCTCTTGAAACGTGACACAGTTGCGCAAG | | |  |
| **p16 shRNA (mouse)** | | | | |
| p16R-1F | GATCCagcgcgcgggccgcccactccTTCAAGAGAGGAGTGGGCGGCCCGCGCGCTTTTTTTG | | |  |
| p16R-1R | AATTCAAAAAAagcgcgcgggccgcccactccTCTCTTGAAGGAGTGGGCGGCCCGCGCGCTG | | |  |
| p16R-2F | GATCCgtgcacgacgcagcgcgggaaTTCAAGAGATTCCCGCGCTGCGTCGTGCACTTTTTTG | | |  |
| p16R-2R | AATTCAAAAAAgtgcacgacgcagcgcgggaaTCTCTTGAATTCCCGCGCTGCGTCGTGCACG | | |  |
| p16R-3F | GATCCtatttgcgttccgctgggtgCTTCAAGAGAGCACCCAGCGGAACGCAAATATTTTTTG | | |  |
| p16R-3R | AATTCAAAAAAtatttgcgttccgctgggtgCTCTCTTGAA GCACCCAGCGGAACGCAAATAG | | |  |
|  |  | | |  |
| **Name** | **Sequence (5’-3’)** | | | **Product size** |
| p16R-4F | GATCCgtgtctagcatgtggctttaaTTCAAGAGATTAAAGCCACATGCTAGACACTTTTTTG | | |  |
| p16R-4R | AATTCAAAAAAgtgtctagcatgtggctttaaTCTCTTGAATTAAAGCCACATGCTAGACACG | | |  |
| NC-F | GATCCTTCTCCGAACGTGTCACTTCAAGAGAGTGACACGTTCGGAGAATTTTTTG | | |  |
| NC-R | AATTCAAAAAATTCTCCGAACGTGTCACTCTCTTGAATGACACGTTCGGAGAAG | | |  |
| **Pitx2 shRNA (mouse)** | | | | |
| PR-1F | GATCCGCCTGAATAACTTGAACAACCTTCAAGAGAGGTTGTTCAAGTTATTCAGGCTTTTTTG | | |  |
| PR-1R | AATTCAAAAAAGCCTGAATAACTTGAACAACCTCTCTTGAAGGTTGTTCAAGTTATTCAGGCG | | |  |
| PR-2F | gatccgggccagcaaggaaagaatgattcaagagatcattctttccttgctggcccttttttg | | |  |
| PR-2R | aattcaaaaaagggccagcaaggaaagaatgatctcttgaatcattctttccttgctggcccg | | |  |
| PR-3F | GATCCGGCTATTCGTACAACAATTGGTTCAAGAGACCAATTGTTGTACGAATAGCC TTTTTTG | | |  |
| PR-3R | AATTCAAAAAAGGCTATTCGTACAACAATTGGTCTCTTGAACCAATTGTTGTACGAATAGCCG | | |  |
| PR-4F | GATCCGCATACAATCTCCGATACTTCTTCAAGAGAGAAGTATCGGAGATTGTATGC TTTTTTG | | |  |
| PR-4R | AATTCAAAAAAGCATACAATCTCCGATACTTCTCTCTTGAAGAAGTATCGGAGATTGTATGCG | | |  |
| NC-F | GATCCTTCTCCGAACGTGTCACTTCAAGAGAGTGACACGTTCGGAGAATTTTTTG | | |  |
| NC-R | AATTCAAAAAATTCTCCGAACGTGTCACTCTCTTGAATGACACGTTCGGAGAAG | | |  |
| **Pitx2 shRNA (human)** | | | | |
| hPR-1F | GATCCGCCGACTCCTCCGTATGTTTATTCAAGAGATAAACATACGGAGGAGTCGGCTTTTTTG | | |  |
| hPR-1R | AATTCAAAAAAGCCGACTCCTCCGTATGTTTATCTCTTGAATAAACATACGGAGGAGTCGGCG | | |  |
| hPR-2F | GATCCGAGTCCGGGTTTGGTTCAAGATTCAAGAGATCTTGAACCAAACCCGGACTCTTTTTTG | | |  |
| hPR-2R | AATTCAAAAAAGAGTCCGGGTTTGGTTCAAGATCTCTTGAATCTTGAACCAAACCCGGACTCG | | |  |
| hPR-3F | GATCCGCCTGAGACTGAAAGCAAAGCTTCAAGAGAGCTTTGCTTTCAGTCTCAGGCTTTTTTG | | |  |
| hPR-3R | AATTCAAAAAAGCCTGAGACTGAAAGCAAAGCTCTCTTGAAGCTTTGCTTTCAGTCTCAGGCG | | |  |
| **Name** | **Sequence (5’-3’)** | | | **Product size** |
| hPR-4F | GATCCGGCTATTCCTACAACAACTGGTTCAAGAGACCA | | |  |
|  | GTTGTTGTAGGAATAGCCTTTTTTG | | |  |
| hPR-4R | AATTCAAAAAAGGCTATTCCTACAACAACTGGTCTCTTGAACCAGTTGTTGTAGGAATAGCCG | | |  |
| hNC-F | GATCCTTGCGCAACTGTGTCACGTTTCAAGAGAACGTGACACAGTTGCGCAATTTTTTG | | |  |
| hNC-R | AATTCAAAAAATTGCGCAACTGTGTCACGTTCTCTTGAAACGTGACACAGTTGCGCAAG | | |  |
| **Methylation-specific primer** | | | | |
| Pitx2  (human) | methylation | | F:GGTTTTTATTTTTAGGAGGAGGAC | 115 |
|  |  |  | R:CAAAAAAACTACAATACAATCCGAT |  |
|  | unmethylation | | F: GTTTTTATTTTTAGGAGGAGGATG | 115 |
|  |  |  | R: AAAAAAACTACAATACAATCCAAT |  |
| **ChIP primer** | | | | |
| Pitx2 | F: CTTGCCTGTGTAGACCCAGT | | | 154 |
| (human) | R: CCTCCTCCCAGACCCTTCT | | |  |
| PPP2R2A | F: GGTGCGGACAGAGGGTATG | | | 221 |
| (human) | R: TCCACTTTCAAAATGGCGCC | | |  |
